# Pthlha and mechanical force control early patterning of growth zones in the zebrafish craniofacial skeleton

**DOI:** 10.1242/dev.199826

**Published:** 2022-01-20

**Authors:** Diego J. Hoyle, Daniel B. Dranow, Thomas F. Schilling

**Affiliations:** Department of Developmental and Cell Biology, University of California, Irvine, CA 92693, USA

**Keywords:** *Danio rerio*, Growth zone, Growth plate, Cartilage hypertrophy

## Abstract

Secreted signals in patterning systems often induce repressive signals that shape their distributions in space and time. In developing growth plates (GPs) of endochondral long bones, Parathyroid hormone-like hormone (Pthlh) inhibits Indian hedgehog (Ihh) to form a negative-feedback loop that controls GP progression and bone size. Whether similar systems operate in other bones and how they arise during embryogenesis remain unclear. We show that Pthlha expression in the zebrafish craniofacial skeleton precedes chondrocyte differentiation and restricts where cells undergo hypertrophy, thereby initiating a future GP. Loss of Pthlha leads to an expansion of cells expressing a novel early marker of the hypertrophic zone (HZ), *entpd5a*, and later HZ markers, such as *ihha*, whereas local Pthlha misexpression induces ectopic *entpd5a* expression. Formation of this early pre-HZ correlates with onset of muscle contraction and requires mechanical force; paralysis leads to loss of *entpd5a* and *ihha* expression in the pre-HZ, mislocalized *pthlha* expression and no subsequent ossification. These results suggest that local Pthlh sources combined with force determine HZ locations, establishing the negative-feedback loop that later maintains GPs.

## Abstract (precis)

**Summary:** Embryonic expression of Pthlha precedes Ihha in establishing the negative-feedback loop that regulates endochondral growth zone formation and establishment of this pattern depends on the force of muscle contraction.

## INTRODUCTION

A fundamental question in developmental biology is how cell identities are defined depending on their position within organs and tissues. In classical morphogen models, signaling gradients form through diffusion and induce different cell fates at distinct concentration thresholds (Wolpert, 1969). However, these models fail to address how such a gradient self-regulates. Several embryonic patterning signals regulate their own activity through the induction of a repressive signal, forming negative-feedback loops that often lead to temporal oscillations and spatially periodic patterns of gene expression (Bastida et al., 2009; Lander, 2007; Schier and Shen, 2000; Schilling et al., 2012). However, in most cases the events that initially establish these feedback loops are unknown.

Growth plates (GPs) of endochondral bones in vertebrates are controlled by a negative-feedback loop that coordinates the rate of cartilage proliferation and differentiation to shape the bone that replaces the cartilage. GPs of long bones in the limbs contain three major regions through which chondrocytes transition as they mature: resting zones (RZs) near the distal ends, more proximal proliferating zones (PZs) that drive cartilage growth, and hypertrophic zones (HZs) located in the mid-region where cartilage is replaced by bone. Chondrocytes in HZs exit the cell cycle, swell with vacuoles, secrete large amounts of extracellular matrix (ECM) proteins, such as Collagen 10 (*Col10a1*), express Indian hedgehog (Ihh), and finally undergo apoptosis (Kronenberg, 2003). Secretion of Ihh induces osteoblasts in the surrounding perichondrium, forming a bony collar around the dying hypertrophic chondrocytes (St-Jacques et al., 1999), though recent studies also suggest that some osteoblasts also derive directly from chondrocytes within the HZ that do not undergo apoptosis (Yang et al., 2014a,b). Ihh also acts on neighboring chondrocytes in the RZ to induce Parathyroid hormone-like hormone (Pthlh), which feeds back to inhibit Ihh in HZs via repression of Runt-related transcription factor 2 (Runx2) (Vortkamp et al., 1996). This prevents chondrocyte hypertrophy, promotes proliferation in PZs, and maintains a reserve of chondrocytes in RZs (Chung et al., 1998; Karp et al., 2000; Li et al., 2004; Schipani et al., 1997; St-Jacques et al., 1999; Weir et al., 1996; Yoshida et al., 2004). Loss-of-function mutations in either *PTHLH* or *IHH* in humans cause brachydactyly, characterized by short digits, as well as short stature due to defects in bone length (Gao et al., 2001; Klopocki et al., 2010).

At least some components of this signaling network that controls endochondral bone growth are also regulated by mechanical force. Paralysis of chick embryos dramatically decreases chondrocyte proliferation in the PZ of developing long bone GPs (Germiller and Goldstein, 1997), whereas applying more force to GPs increases chondrocyte proliferation and bone size (Wang and Mao, 2002). Restraining movement also reduces both Pthlh and Ihh expression in mandibular condylar cartilage and at fibrous insertions at entheses (Chen et al., 2007; Jahan et al., 2014; Rais et al., 2015). Cartilage cells likely sense pulling and compressive forces through mechanoreceptors, such as Piezo-type mechanosensitive ion channel component 1 (Piezo1) and Piezo2, as well as transient receptor potential vanilloid 4 (Trpv4), all of which promote chondrocyte differentiation *in vitro* (Muramatsu et al., 2007; O'Conor et al., 2014; Servin-Vences et al., 2017). Compressive forces applied to mid-palatal suture chondrocytes in rats increase Col10a1 expression and cartilage hypertrophy (Saitoh et al., 2000). This influence of force on gene expression in mature GPs suggests that mechanical force may also regulate GP formation during embryogenesis.

Cartilages in the embryonic and larval zebrafish provide a relatively simple system for exploring early GP and growth zone (GZ) formation as well as the effects of mechanical force (Le Pabic et al., 2014; Heubel et al., 2021). Most zebrafish cartilages start out as a few linear rows of chondrocytes, with several orders of magnitude fewer cells than their mammalian counterparts (Schilling and Kimmel, 1997). The ceratohyal (ch) cartilage is a rod-shaped cartilage that supports the jaw and has a primary ossification center in the middle that forms in the embryo, and secondary ossification centers near the ends that form in larvae as they age, a linear organization that resembles GPs in mammalian long bones (Albertson et al., 2010; Brinkley et al., 2016). However, unlike its mammalian counterpart, the ch cartilage does not proliferate during the initial stages of hypertrophic differentiation (Kimmel et al., 1998). This enables testing of the effects of signals and mechanical forces on hypertrophic differentiation largely independent of the effects on chondrocyte proliferation that may affect the speed by which chondrocytes leave the influence of Pthlh and become hypertrophic. HZs in zebrafish cartilages closely resemble mammalian HZs (Eames et al., 2010, 2011; Mitchell et al., 2013). Zebrafish have two *Pthlh* orthologs, *pthlha* and *pthlhb*, expressed in RZs and two Ihh orthologs, *ihha* and *ihhb*, expressed in HZs. *pthlha* is more similar to mammalian *Pthlh* in its sequence, expression pattern and loss-of-function phenotype (Yan et al., 2012).

Previous studies have argued that the Pthlh-Ihh feedback loop in long bones of tetrapod limbs is not active until later in GP development based on a lack of detectable *Pth1r* expression in chondrocytes (Vortkamp et al., 1996). Here, we show that the transgene *entpd5a:kaede* marks chondrocytes in pre-HZs of the fully formed ch cartilage of zebrafish embryos, preceding the previously reported onset of *ihha* or *col10a1* expression by 2 days (Eames et al., 2010), and is regulated by Pthlha. *pthlha* expression is initiated at least a day earlier in dorsal and ventral domains of cartilage progenitors that flank the future site of *entpd5a:kaede* expression, potentially restricting the pre-HZ to its central position. Consistent with this hypothesis, we observed that loss of *pthlha* expression leads to an increase in the number of *entpd5a:kaede*-expressing chondrocytes, expanding the pre-HZ domain outside of its normal zone within the cartilage. Conversely, mosaic ectopic *pthlha* expression in subsets of cells within the ch cartilage disrupts pre-HZ formation depending on their proximity to one another and can induce ectopic HZs. In addition, we show that mechanical force is required for presumptive pre-HZ formation, as paralysis leads to loss of *pthlha* localization and early *entpd5a:kaede* expression as well as later reductions in *ihha* expression in ch cartilages. These results suggest that localized expression of Pthlh determines the location of HZs within the ch, initiating the negative-feedback loop with Ihh that persists into adulthood, and that this formation of the rudiments of an early embryonic GP requires mechanical force.

## RESULTS

### *entpd5a:kaede* expression marks early hypertrophic chondrocytes

**Fig. 1.**
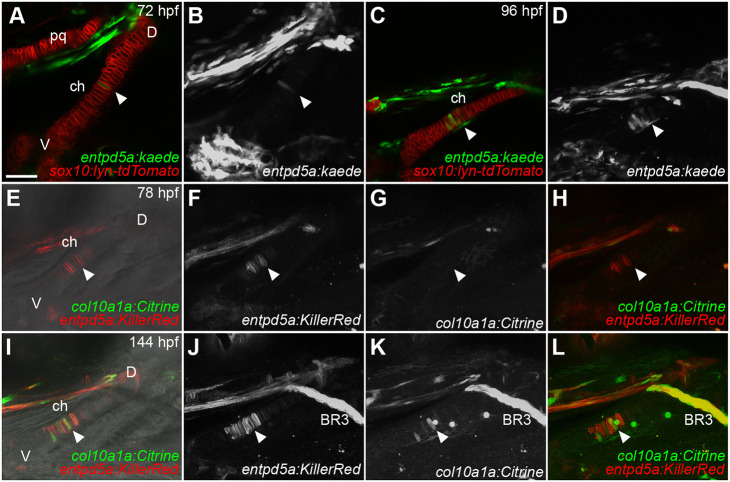
**Pre-hypertrophic ceratohyal (ch) chondrocytes express *entpd5a:kaede* soon after differentiation.** Confocal images of live double-transgenic embryos. (A-D) *sox10:lyn-tdtomato;entpd5a:kaede* double-transgenic embryos at 72 hpf (A,B) and 96 hpf (C,D). (E-L) *col10a1a:Citrine;entpd5a:KillerRed* double-transgenic embryos at 78 hpf (E-H) and 144 hpf (I-L). (E,I) Optical slices in DIC. (A,C,E,I) Optical slices. (B,D,F-H,J-L) *z*-projections. White arrowheads indicate the position of the pre-HZ. BR3, branchiostegal ray 3; ch, ceratohyal; D, dorsal; pq, palatoquadrate; V, ventral. All micrographs are ventral views with anterior to the left. Scale bar: 50 μm (A-L).

Hypertrophic chondrocytes of mature GPs in mammals express *Col10a1* and *Ihh* (Girkontaite et al., 1996; Vortkamp et al., 1996). The zebrafish *col10a1a:Citrine* transgene marks hypertrophic chondrocytes in the ch cartilage starting at 120 hours post-fertilization (hpf) and both *ihha* and *ihhb* expression are initiated in these cells slightly later (Eames et al., 2010; Mitchell et al., 2013). Surprisingly, we found that the *entpd5a:kaede* bacterial artificial chromosome (BAC) transgene, which had previously been reported to be expressed in osteoblasts during development and wound repair (Geurtzen et al., 2014; Lleras Forero et al., 2018; Lopez-Baez et al., 2018), was expressed in chondrocytes at the center of the ch cartilage at 72 hpf, in a region similar to *Col10a1* expression, but 2 days earlier, soon after the chondrocytes differentiate (Fig. 1). Entpd5a regulates phosphate homeostasis during osteogenesis and *entpd5a^−/−^* mutant zebrafish lack bone, but potential indirect roles in cartilage hypertrophy have not been addressed (Huitema et al., 2012).

To determine the identities of these *entpd5a:kaede^+^* chondrocytes as potentially pre-hypertrophic, we generated *sox10:lyn-tdTomato;entpd5a:kaede* double-transgenic embryos, in which chondrocyte membranes were labeled in red, and looked for co-expression with *entpd5a:kaede* (cytoplasmic, green) in the ch cartilage from 72 to 96 hpf. A few *entpd5a:kaede*^+^ chondrocytes were detected in the center of the stack of ch chondrocytes at 72 hpf (Fig. 1A,B). The number increased by 96 hpf (Fig. 1C,D) and their positions correlated with that of *col10a1* and *ihha* expression (Eames et al., 2010; Mitchell et al., 2013). To confirm their identities, we generated *entpd5a:KillerRed;col10a1a:Citrine* double-transgenic embryos and looked for co-expressing cells within the ch cartilage. *entpd5a:KillerRed* was detected in a few chondrocytes at 78 hpf but *col10a1a:Citrine* was not (Fig. 1E-H). However, by 144 hpf the number of *entpd5a:KillerRed*-expressing cells had increased, and a few *col10a1a:Citrine* expressing cells were present within the same zone, with some cells co-expressing both markers (Fig. 1I-L). These results suggest that *entpd5a* precedes *col10a1a* expression in pre-hypertrophic chondrocytes in ch cartilage, which later become part of HZs.

### *pthlha* expression precedes *ihha* expression and is required for initiation of cartilage hypertrophy

**Fig. 2.**
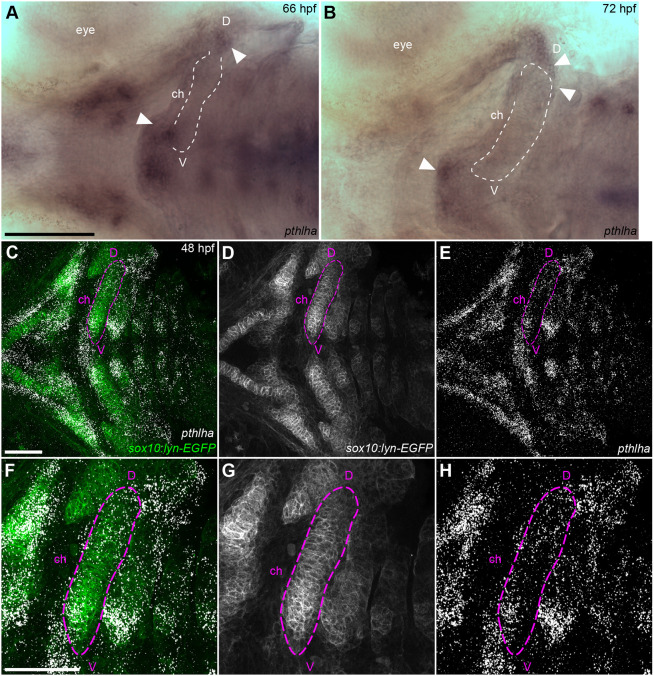
***pthlha* is expressed in dorsal and ventral subdomains of ch cartilage progenitors prior to differentiation.** (A,B) *In situ* hybridization for *pthlha* mRNA at 66 hpf (A) and 72 hpf (B). The ch cartilage is outlined. White arrowheads indicate *pthlha* expression zones. (C-H) *pthlha* HCR in *sox10:lyn-EGFP;entpd5a:KillerRed* double-transgenic embryos at 48 hpf. F-H show magnified views of the outlined ch cartilage. *entpd5a:KillerRed* is not detected at 48 hpf. Ventral views, anterior to the left. ch, ceratohyal; D, dorsal; V, ventral. Scale bars: 100 μm (A,B); 50 μm (C-H).

**Fig. 3.**
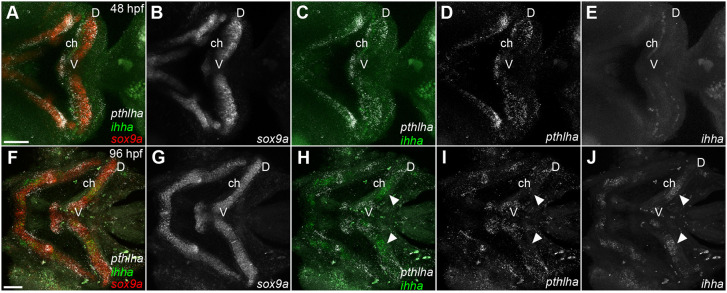
***pthlha* expression in cartilage progenitors precedes expression of *ihha*.** (A-J) HCR *in situ* hybridization for *pthlha*, *ihha* and *sox9a* in wild-type embryos at 48 hpf (A-E) and 96 hpf (F-J). White arrowheads indicate the ceratohyal *ihha* expression domain. All micrographs are ventral view *z*-projections with anterior to the left. ch, ceratohyal; D, dorsal; V, ventral. Scale bar: 50 μm (A-J).

In tetrapod long-bone GPs, *Pthlh* expressed in the RZ prevents expansion of the HZ, thereby controlling its size (Chung et al., 1998; Schipani et al., 1997). The zebrafish *Pthlh* ortholog, *pthlha*, is expressed in developing craniofacial cartilages prior to the onset of ossification and is required for the appropriate timing of ossification (Yan et al., 2012). However, the locations of *pthlha* expression with respect to forming HZs in these cartilages have not been examined. We performed whole-mount *in situ* hybridization for *pthlha* in zebrafish and found that, as early as 66 hpf, *pthlha* expression was restricted to regions near the ventral and dorsal ends of the ch cartilage and other pharyngeal cartilages (Fig. 2A,B), flanking a mid-region marked by *entpd5a:kaede* expression several hours later. Hybridization chain reaction (HCR) *in situ* hybridizations for *pthlha* carried out in *sox10:lyn-GFP;entpd5a:KillerRed* double-transgenic embryos to mark chondrocytes and hypertrophic cells, detected a similar *pthlha* expression pattern even earlier, at 48 hpf, in precursor cells within the cartilage condensation that forms the ch cartilage (Fig. 2C-E). We did not detect any *entpd5a:KillerRed* signal in precursor cells at 48 hpf. HCRs performed later, at 96 hpf, revealed that *pthlha* expression persisted and became increasing localized within and around the ventral and dorsal ends of the ch cartilage (Fig. 3). Expression of the Pthlh receptor *pth1ra* was widespread throughout cartilage and surrounding tissues (data not shown). Zebrafish also have a *pthlha* paralog, *pthlhb*, and we examined its expression at 72 hpf with HCR but did not detect expression in the ch cartilage at this stage (Fig. S1).These results suggest that *pthlha* expression prefigures the locations of future craniofacial GPs, and acts at a distance to determine sites of HZ formation.

Ihh in GPs of mammalian long bones is expressed in pre-hypertrophic and hypertrophic chondrocytes (HCs), and HZ formation is delayed in mutants in which Hh signaling is disrupted (Long et al., 2001; St-Jacques et al., 1999). The zebrafish orthologs *ihha* and *ihhb* are expressed in the developing ch cartilage at 120 hpf (Eames et al., 2010). Using HCR, we detected *ihha* and *ihhb* expression in the future HZ at 72 and 96 hpf, but did not detect them at earlier stages (Fig. 3; Fig. S1). To determine whether *entpd5a* expression in putative pre-HZs requires Hh signaling, we used the Smoothened (Smo) antagonist cyclopamine (CyA) to treat *sox10:lyn-tdTomato;entpd5a:kaede* double-transgenic embryos from 72-96 hpf and examined the number of *entpd5a:kaede*-labeled chondrocytes at 120 hpf (Fig. S2A-D). Whereas mock-treated embryos had an average of 9.9 *entpd5a:kaede*-labeled chondrocytes (*n*=10), CyA-treated embryos had significantly fewer kaede-positive cells (1.8/embryo; *n*=8; *P*=0.016) indicating that *entpd5a:kaede* expression requires Hh signaling, similar to mature HZs (Fig. S2E).

Null *Pthlh^−/−^* mutant mice exhibit increased and premature ossification, reduced chondrocyte proliferation and postnatal lethality (Karaplis et al., 1994). Similarly, morpholino (MO) knockdown of zebrafish *pthlha* leads to increased endochondral ossification (Yan et al., 2012), suggesting functional conservation between zebrafish *pthlha* and mouse *Pthlh*. However, this is unlikely to represent a complete loss of *pthlha* during skeletogenesis because MOs typically lose effectiveness at the later stages of embryonic development in zebrafish (>60 hpf). In order to determine the requirements for *pthlha* in HZ formation, we took advantage of a recent method for efficient CRISPR-Cas9 F0 mutagenesis (Wu et al., 2018) to generate *pthlha* F0 mutants in the *sox10:lyn-tdTomato;entpd5a:kaede* double-transgenic background. CRISPR-induced cutting was confirmed by heteroduplex mobility shift assays (Fig. S3). CRISPR-induced deletions were confirmed phenotypically in injected embryos by the early onset of ossification of *entpd5a:kaede*-labeled bone around the ch cartilage.

**Fig. 4.**
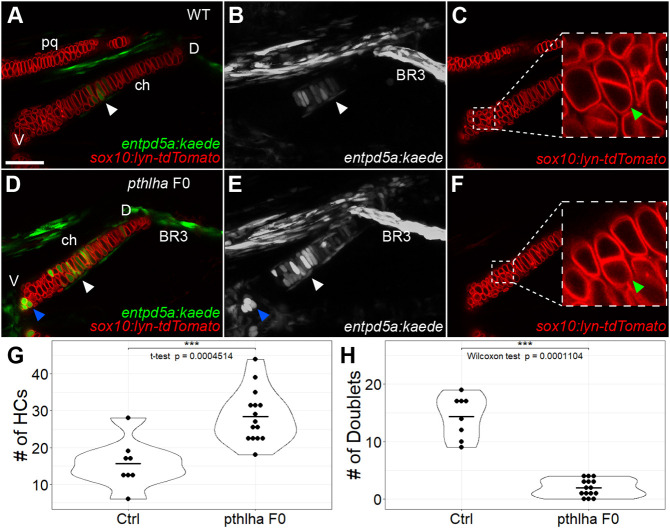
**Loss of *pthlha* leads to dorsal expansion of *entpd5a*-expressing pre-hypertrophic chondrocytes in ch cartilage.** (A-C) Live imaging of wild-type *sox10:lyn-tdTomato;entpd5a:kaede* double-transgenic embryos; ventral views. (D-F) Live imaging of *pthlha* CRISPR F0 *sox10:lyn-tdTomato;entpd5a:kaede* double-transgenic embryos; ventral views. (C,F) Optical slices showing the *sox10:lyn-tdTomato* chondrocyte doublets, magnified in insets*.* (A,C,D,F) Optical slices, (B,E) *z*-projections. White arrowheads indicate the position of the HZ. Blue arrowheads indicate the position of the secondary HZ. Green arrowheads indicate the position of chondrocyte doublets. (G) Quantification of *entpd5a:kaede*-labeled pre-hypertrophic chondrocytes (HCs) in the ch cartilage. (H) Quantification of chondrocyte doublets in the ch cartilage. BR3, branchiostegal ray 3; ch, ceratohyal; Ctrl, control; D, dorsal; pq, palatoquadrate; V, ventral. Scale bar: 50 μm (A-F).

We examined changes in the numbers and locations of *entpd5a:kaede-*labeled chondrocytes at 120 hpf in the absence of Pthlha function. Whereas CRISPR-Cas9 non-injected controls had 15.6 *entpd5a:kaede-*labeled chondrocytes on average (*n*=8), *pthlha* F0 mutants had nearly twice as many (28.5/embryo; *n*=15; *t*-test *P*=0.0005) (Fig. 4A,B,D,E,G). The zone of *entpd5a:kaede-*labeled chondrocytes expanded dorsally along the ch cartilage in *pthlha* F0 mutants, as evident from the presence of *entpd5a:kaede-*labeled chondrocytes and perichondrial osteoblasts much closer to branchiostegal ray 3 (BR3), than in non-injected controls (Fig. 4B,E). BR3 is a dermal bone formed independently of cartilage that extends away from the dorsal edge of the ch cartilage. These results suggest that *pthlha* is required to dorsally restrict the locations of hypertrophic chondrocytes in the ch cartilage.

**Fig. 5.**
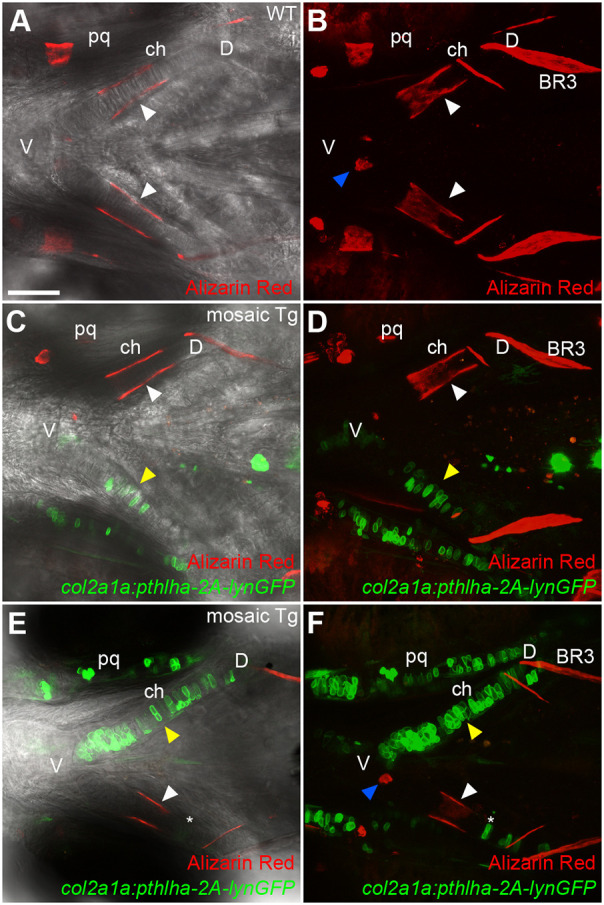
**Mosaic *pthlha* misexpression in ch cartilage disrupts ch ossification patterns.** (A-F) Live imaging of 144 hpf wild-type (A,B) and *col2a1a:pthlha-2A-lynGFP* mosaic transgenic (C-F) embryos treated with Alizarin Red. (A,C,E) Optical slices in DIC. (B,D,F) *z*-projections. White arrowheads indicate the position of the HZ. Yellow arrowheads indicate the position of *col2a1a:pthlha-2A-lynGFP*-expressing cells. Blue arrowheads indicate the position of the secondary hypertrophic HZ. Asterisks indicate the position of a *col2a1a:pthlha-2A-lynGFP*-labeled cell disrupting bone collar formation. BR3, branchiostegal ray 3; ch, ceratohyal; D, dorsal; pq; palatoquadrate; V, ventral. All micrographs are ventral views with anterior to the left. Scale bar: 50 μm (A-F).

Interestingly, *pthlha* CRISPR F0 mutants also had a few *entpd5a:kaede*-labeled chondrocytes at the ventral end of the ch cartilage at 120 hpf, which was almost never seen in controls (Fig. 4A,B,D,E), suggestive of a premature ventral secondary HZ. The ch cartilage in juvenile zebrafish, like mammalian long bones, has primary ossification zones in the mid-region along its long axis and secondary ossification zones at the ends. The ventral secondary zone is first visible in Alizarin Red-stained embryos at 144 hpf (Fig. 5A,D), whereas the dorsal zone stains with Alizarin Red at approximately 1 month of age, similar to ch cartilages of sticklebacks (Albertson et al., 2010). All *pthlha* CRISPR F0 mutants had an *entpd5a:kaede*-free zone between the center and the ventral end of the cartilage (Fig. 4D,E), suggesting that loss of Pthlh signaling leads not only to expansion of the primary pre-HZ but also to early onset of a secondary set of hypertrophic chondrocytes.

In addition to the obvious increase in *entpd5a:kaede*-labeled chondrocyte number, the ch cartilage in *pthlha* F0 mutants also appeared approximately 20% shorter in length (Fig. 4A,D). Because *Pthlh* mutant mice have reduced proliferation (Karaplis et al., 1994), we examined proliferation rates in *pthlha* F0 mutants. Chondrocytes secrete large amounts of ECM, which encapsulates and separates them from each other. However, chondrocytes that have just undergone cytokinesis do not have ECM in between their cellular membranes and appear as symmetrical ‘doublets’. We visualized these doublets in *sox10:lyn-tdTomato* transgenics and found them largely restricted to the ventral portion of the ch cartilage, with 14.8 doublets on average (*n*=8) in uninjected controls. In contrast, the number of doublets was dramatically reduced in *pthlha* F0 mutants (1.9/embryo; *n*=15; *P*=0.0001) (Fig. 4C,F,H).

However, reduced ch cartilage size and proliferation in *pthlha*-mutant embryos could simply reflect developmental delay. To control for this, we examined ossification of BR3, because it is a dermal bone attached to the ch cartilage that expresses *entpd5a* and forms independently of cartilage via direct ossification of mesenchymal progenitors, it ossifies at the same stages and it is largely insensitive to Ihh and Pthlh (Felber et al., 2011). At 72 hpf, an occasional *entpd5a:kaede*-labeled BR3 progenitor was detected in controls (Fig. S4A). By 96 hpf, Alizarin Red live staining of double-transgenic embryos revealed mineralized bone matrix in BR3 between the osteoblasts marked by *entpd5a:kaede* (Fig. S4D,E) as well as *entpd5a:kaede*-labeled cells in the pre-HZ region of the ch cartilage (Fig. S4F) and BR3 continued to grow and ossify over the next 2 days (Fig. S4B,C). The BR3 appeared indistinguishable between control and *pthlha* F0 mutants (Fig. 4B,E), suggesting that controls and F0 mutants were stage-matched. Together, these results suggest that *pthlha* plays roles both in patterning HZs and in cartilage proliferation.

### Mosaic misexpression of *pthlha* in cartilage disrupts pre-HZ patterning

Targeted expression of human *PTHLH* in mouse cartilages using a *Col2a1* promoter delays HZ formation as well as ossification (Weir et al., 1996). We hypothesized that ectopic expression of *pthlha* in localized zones within the embryonic ch cartilage in zebrafish would disrupt hypertrophy and ossification at a local level. To test this, we generated a fusion construct to co-express *pthlha* and *lyn-GFP* polycistronically under the control of the zebrafish *col2a1a* promoter. DNA encoding this *col2a1a:pthlha-2A-lynGFP* construct was injected to generate mosaic transgenic F0 zebrafish. These were assessed for effects of localized *pthlha* expression, marked by *lyn-GFP* expression, on ossification (i.e. formation of a bone collar) around the primary HZ. Ossification occurred normally in non-injected 144 hpf embryos, as assessed by live Alizarin Red staining (Fig. 5A,B). However, mosaic transgenic ch cartilages with ectopic *pthlha* expression in the mid-region of the cartilage lacked bone collars (Fig. 5C-F), whereas they formed normally around contralateral ch cartilages, serving as an internal control. In addition, ectopic *pthlha* expression did not disrupt formation of dermal bones, such as BR3 associated with ch (Fig. 5D,F). Interestingly, a few ectopic *pthlha*-expressing cells located on the dorsal side of the ch cartilage disrupted bone collar formation such that the bone collar still formed but appeared shortened from the dorsal side (Fig. 5E,F) compared with undisturbed bone collars (Fig. 5A-D), suggesting that the degree of bone collar disruption is dependent on the number of ectopic *pthlha*-expressing cells and their proximity to the middle of the cartilage where the primary HZ normally forms. Altogether, these results suggest that cells expressing ectopic *pthlha* can locally inhibit ossification in a concentration-dependent manner.

**Fig. 6.**
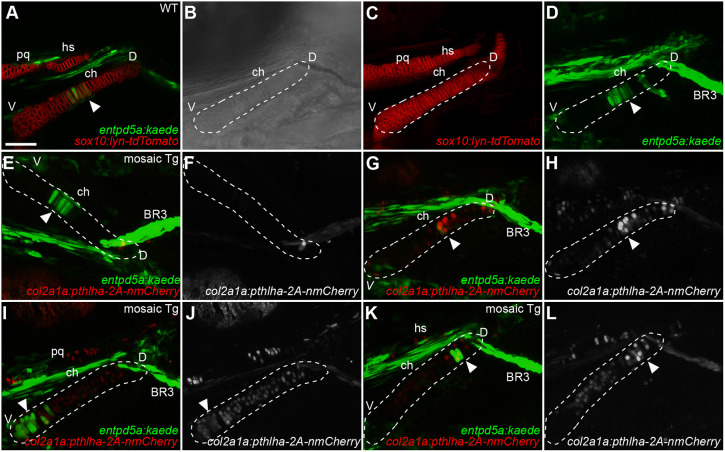
**Mosaic *pthlha* misexpression in ch cartilage disrupts localization of *entpd5a*-expressing pre-hypertrophic chondrocytes.** (A-D) Live imaging of a wild-type *sox10:lyn-tdTomato;entpd5a:kaede* double-transgenic embryo. (B) DIC channel, used to draw an outline of the ch cartilage (dashed line). (E-L) Live imaging of *entpd5a:kaede* transgenic embryos injected with the *col2a1a:pthlha-2A-nmCherry* construct. (E-H) Example of a single embryo with no *col2a1a:pthlha-2A-nmCherry* expression on one side of the ch cartilage (E,F) where *entpd5a:kaede* in the HZ is undisrupted, and the transgenic side of the same embryo (G,H) with *col2a1a:pthlha-2A-nmCherry* expression disrupting *entpd5a:kaede* expression in the HZ. (I,J) Example of another injected embryo in which *col2a1a:pthlha-2A-nmCherry* expression moves the HZ to the ventral part of the ceratohyal. (K,L) Another mosaic transgenic embryo in which *col2a1a:pthlha-2A-nmCherry* expression moves the HZ to the dorsal part of the ceratohyal. White arrowheads indicate the position of the HZ. BR3, branchiostegal ray 3; ch, ceratohyal; D, dorsal; hs, hyosymplectic; pq, palatoquadrate; V, ventral. (A,E,G,I,K) Optical slices. (C,D,F,H,J,L) *z*-projections. All micrographs are ventral views with anterior to the left. Scale bar: 50 μm (A-L).

To determine whether such ectopic *pthlha* expression also disrupts pre-HZ patterning, we replaced *lyn-GFP* in our polycistronic construct with nuclear-localized mCherry (nmCherry), then tested the effects of mosaic ectopic *pthlha* on *entpd5a:kaede* expression. Because *entpd5a:kaede* expression is not limited to cartilage, brightfield optics were used to draw outlines of ch cartilages, and hypertrophic chondrocytes were localized within the outlines (Fig. 6A-D). In mosaic transgenic F0 zebrafish at 120 hpf, whereas *entpd5a:kaede*-labeled clusters of hypertrophic chondrocytes formed normally in ch cartilages that did not integrate the transgene and express ectopic *pthlha* (Fig. 6E,F), they were reduced or completely absent in contralateral ch cartilages that expressed ectopic *pthlha* in the vicinity of the future pre-HZ (Fig. 6G,H). These results suggest that Pthlha inhibits both embryonic cartilage hypertrophy and ossification.

Interestingly, in some cases when ectopic *pthlha* expression was widespread throughout much of the ch cartilage, ectopic *entpd5a:kaede*-labeled cells were detected either on the ventral (Fig. 6I,J) or dorsal (Fig. 6K,L) ends of the cartilage element. In all these cases, at least a few of these ectopic hypertrophic chondrocytes expressed ectopic *pthlha*. Similarly, chondrocytes expressing ectopic *PTHLH* or constitutively active *PTH1R* in mice become hypertrophic (Schipani et al., 1997; Weir et al., 1996). However, in contrast to the results in mice, ectopic hypertrophic chondrocytes induced by Pthlha in zebrafish were not associated with ectopic ossification. Altogether, these results suggest that Pthlh restricts hypertrophic chondrocytes to the mid-cartilage region, thereby determining pre-HZ position along the length of the cartilage. These results also suggest that initiation of cartilage hypertrophy is both spatially and temporally regulated by local exposure to Pthlh signaling.

### Paralysis prevents pre-HZ formation prior to the onset of proliferation

**Fig. 7.**
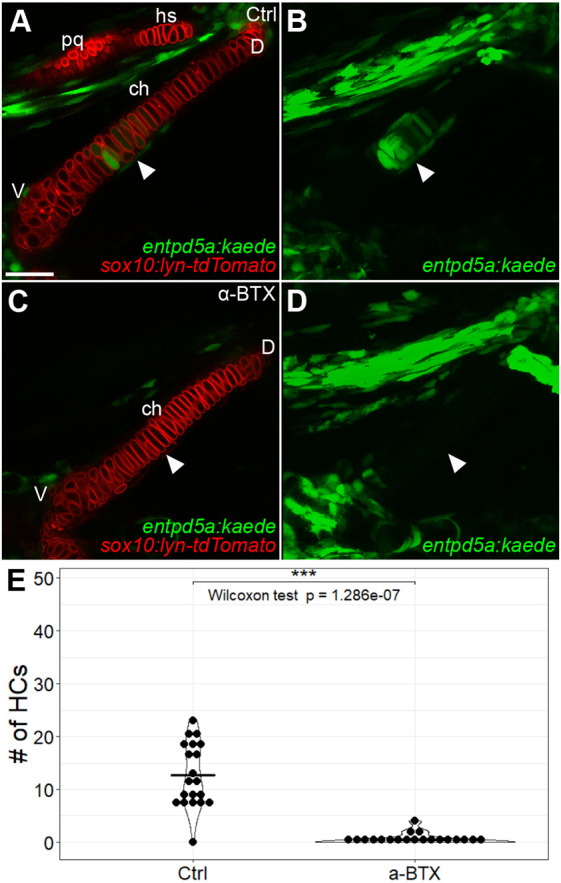
**Early paralysis reduces numbers of *entpd5a*-expressing pre-hypertrophic chondrocytes in ch cartilage.** (A-D) Live imaging of wild-type control (A,B) and α-BTX-injected paralyzed (C,D) *sox10:lyn-tdTomato;entpd5a:kaede* double-transgenic embryos at 96 hpf. The HZ does not develop in α-BTX-injected animals. (A,C) Optical slices. (B,D) *z*-projections. White arrowheads indicate the position of the HZ. (E) Quantification of number of *entpd5a:kaede*-labeled chondrocytes in the ch cartilage. ch, ceratohyal; D, dorsal; hs, hyosymplectic; pq, palatoquadrate; V, ventral. All micrographs are ventral views with anterior to the left. Scale bar: 50 μm (A-D).

Previous studies have shown that mechanical force influences GP dynamics. Though effects of force on cartilage hypertrophy have previously been reported (Nowlan et al., 2008; Saitoh et al., 2000; Wang and Mao, 2002), these studies were carried out in model organisms in which chondrocytes proliferate. This makes it difficult to distinguish between potential direct effects of force on the signaling mechanisms controlling hypertrophic differentiation versus changes in proliferation that alter the number of hypertrophic chondrocytes as their proximity to the source of Pthlh changes. Craniofacial cartilages in zebrafish present an opportunity to investigate proliferation-independent effects on hypertrophy because chondrocytes do not proliferate until after 96 hpf (Kimmel et al., 1998). *entpd5a* expression appears in pre-hypertrophic chondrocytes in the ch cartilage at 72 hpf, and this coincides with the onset of jaw movements. To test the hypothesis that force regulates initiation of *entpd5a* expression in these cells, we paralyzed *sox10:lyn-tdTomato;entpd5a:kaede* double-transgenic embryos starting from 68 hpf by injecting alpha bungarotoxin (α-BTX) protein, an acetylcholine receptor antagonist, into the bloodstream, and examined the number of *entpd5a:kaede^+^* cells in the ch cartilage at 96 hpf (Fig. 7A-D). Whereas non-injected embryos had 12.70 *entpd5a^+^* cells on average (*n*=20), α-BTX-injected embryos had very few to none (0.45/embryo, *n*=20; *P*=1.286e−07), suggesting that onset of hypertrophy in embryonic cartilages requires mechanical force (Fig. 7E).

**Fig. 8.**
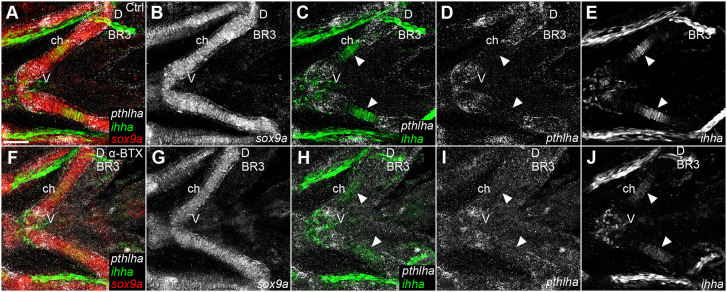
**Early paralysis leads to a reduction in *ihha* expression in pre-hypertrophic chondrocytes and redistribution of *pthlha*.** (A-J) Wild-type control (A-E) and α-BTX-injected paralyzed (F-J) *entpd5a:kaede* transgenic embryos at 96 hpf. All micrographs are ventral view *z*-projections with anterior to the left. White arrowheads indicate the ceratohyal *ihha* expression zones. BR3, branchiostegal ray 3; ch, ceratohyal; D, dorsal; V, ventral. Scale bar: 50 μm (A-J).

To determine whether the mechanical force of muscle contraction regulates *pthlha* and/or *ihha* expression in ch cartilage, we performed HCR *in situ* hybridizations for *pthlha* and *ihha* in paralyzed embryos at 96 hpf (Fig. 8). We found that paralysis induced by α-BTX injections at 72 hpf disrupted the spatial localization of *pthlha* such that it was more evenly distributed along the ch cartilage, as well as striking reductions in *ihha* expression in HZ (Fig. 8I,J). These results indicate that changes in numbers of *entpd5a^+^* cells in the pre-HZ as well as later skeletal defects are secondary to changes in *pthlha* and *ihha* expression.

The ch cartilages in paralyzed embryos were also slightly shorter than those of non-injected embryos, raising the possibility that the loss of *entpd5a*-labeled hypertrophic chondrocytes was simply due to developmental delay. Therefore, we monitored BR3 development in control and α-BTX-injected *sox10:lyn-tdTomato;entpd5a:kaede* double-transgenic embryos (Fig. S4G-I). BR3 appeared virtually identical to controls in *sox10:lyn-tdTomato;entpd5a:kaede* siblings injected with α-BTX at 68 hpf and subsequently stained live with Alizarin Red, despite the absence of *entpd5a:kaede*-labeled chondrocytes, indicating they were stage-matched.

**Fig. 9.**
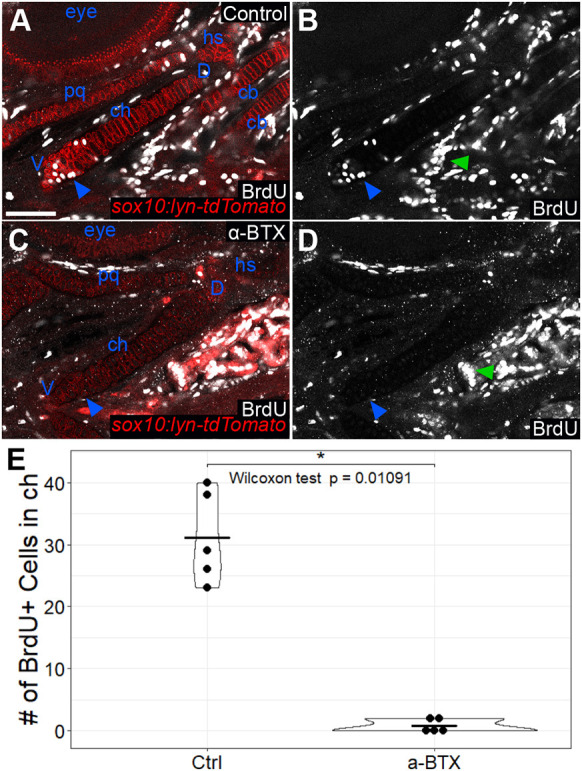
**Later paralysis leads to reductions in chondrocyte proliferation.** (A-D) Anti-BrdU antibody staining of wild-type (A,B) and α-BTX-injected paralyzed (C,D) *sox10:lyn-tdTomato* transgenic embryos at 120 hpf. BrdU staining is absent from cartilages, but not from other tissues in α-BTX-injected animals. Blue arrowheads indicate the ventral aspect of the ch cartilage where chondrocyte proliferation appears to concentrate. Green arrowheads indicate proliferation in tissues other than cartilage. All micrographs are ventral view optical slices with anterior to the left. (E) Quantification of BrdU-labeled cells in the ch cartilage. cb, ceratobranchial; D, dorsal; ch, ceratohyal; hs, hyosymplectic pq, palatoquadrate; V, ventral. Scale bar: 50 μm (A-D).

To determine whether mechanical force promotes proliferation in zebrafish craniofacial cartilages at later stages (after 96 hpf), we monitored proliferation with bromodeoxyuridine (BrdU) labeling (Fig. 9). First, we injected *sox10:lyn-tdTomato* transgenic embryos at 68 hpf with α-BTX, which paralyzed them for approximately 48 h, after which movement recovered. We then treated injected and non-injected embryos with BrdU from 96 to 120 hpf, fixed them at 120 hpf, and performed anti-BrdU antibody staining. By counting the number of BrdU^+^/*sox10:lyn-tdTomato*-labeled cells, we found that most BrdU-labeled chondrocytes in non-injected embryos were restricted to the ventral third of the ch cartilage, with a few in the dorsal tip and occasionally one or two near the middle where the HZ develops (Fig. 9A,B). However, whereas non-injected embryos had 31.2 BrdU-labeled chondrocytes in the ch cartilage on average (*n*=5), α-BTX injected embryos had many fewer (0.8/embryo; *n*=5; Wilcoxon test, *P*=0.011) (Fig. 9). In addition, paralysis led to a complete elimination of BrdU incorporation in several other cartilages (e.g. palatoquadrate, hyomandibular, symplectic), but not in the surrounding muscle or central nervous system. These results suggest that the force produced by muscle contraction promotes proliferation in zebrafish craniofacial cartilages, similar to skeletal GPs in other species (Hu and Albertson, 2017; Wang and Mao, 2002). These changes are likely secondary to changes in *pthlha* and *ihha* expression induced by force.

## DISCUSSION

### An embryonic prepattern shapes craniofacial cartilage GPs and ossification patterns

**Fig. 10.**
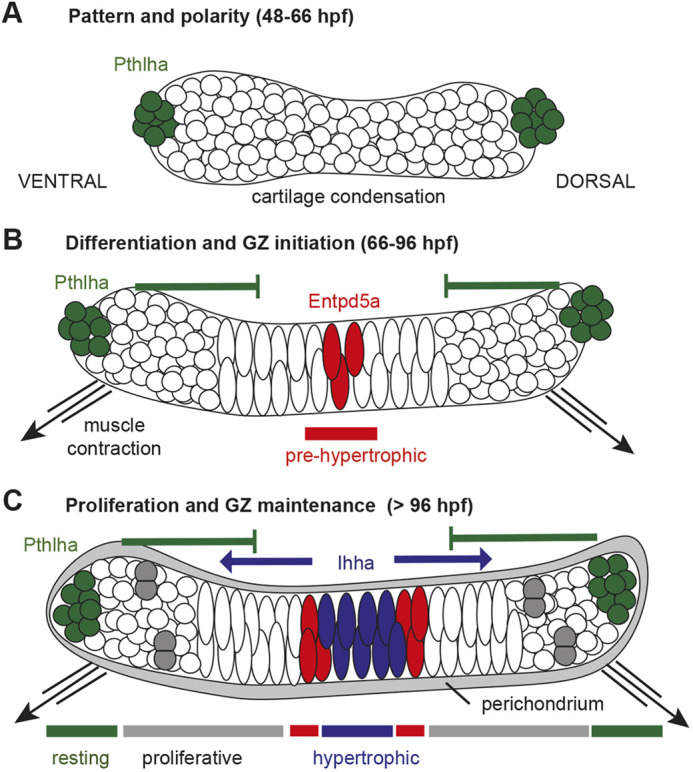
**Model for growth zone establishment in the zebrafish ceratohyal cartilage.** (A-C) Diagram of GZ development in a single ch cartilage on one side of the head as chondrocytes condense (A), differentiate (B) and begin to proliferate (C); ventral to the left. (A) Pthlha-expressing cells (green) are first detected at either end of the developing cartilage condensation at 48-66 hpf. (B) Entpd5a-expressing cells (red) are first detected near the center of the differentiating chondrocyte stack at 66-72 hpf, correlating with the onset of cranial muscle contraction (black arrows). (C) A mature GZ including Ihha expression (blue) acting in opposition to Pthlha (green); cartilage proliferation (gray doublets) and a distinct perichondrium (gray margin) emerge after 96 hpf.

Using a much earlier marker for pre-hypertrophic chondrocytes than previously reported, we show that pre-HZs are specified in the zebrafish ch cartilage soon after chondrocyte differentiation, and that their formation requires both Pthlha and mechanical force. We suggest a model in which Pthlha, along with the forces of muscle contraction, determines timing and spatial distribution of HZs to establish the future GPs that persist into adulthood (Fig. 10). These results are consistent with previous studies showing that a Pthlh/Ihh feedback loop at later stages in the tetrapod limb maintains spatial patterns of GPs in endochondral long bones and that Pthlh signaling controls the rate of hypertrophic differentiation in HZs to maintain a pool of proliferating cells (PCs) (Chung et al., 1998; Schipani et al., 1997; Vortkamp et al., 1996; Weir et al., 1996). However, in contrast to these studies, we find that Pthlh signaling in ch cartilage restricts the first chondrocytes that enter the pre-hypertrophic state to the center of the ch cartilage, thereby specifying the location of the pre-HZ and initiating GP polarity.

In our model, this pre-HZ specified by Pthlh signaling gives rise to the domain of emerging *ihh* expression (Fig. 10), which we show is present at 72 hpf, as much as 24 h later than we first see localized expression of *pthlha*. This early expression of *ihha* and *ihhb* in the ch cartilage suggests the presence of the pre-HZ 2 days earlier than previously reported (Eames et al., 2010). In turn, Ihh from the HZ promotes expression of Pthlh, which provides negative feedback to ensure that the HZ expands slowly and that additional HZs do not form as the cartilage grows (Fig. 10C).

Our model relies on the fact that Pthlha, the inhibitory signal, is expressed first, prior to cartilage differentiation (Fig. 10A), and delineates where the activator signal, Ihh, is expressed after differentiated chondrocytes initiate hypertrophic differentiation to establish the feedback loop (Fig. 10B,C). The factors that specify the localized expression domains of *pthlha* remain to be determined and is an interesting avenue for further investigation. Once established, the longer range of the Pthlh inhibitory signal ensures that expression of the Ihh activator signal remains restricted (Fig. 10C), and this sequence of events may be a common feature of the establishment of negative-feedback loops that regulate growth and differentiation in other contexts.

Several lines of evidence support the hypothesis that *entpd5a:kaede* marks pre-HZs, at least in the ch cartilage. First, some *entpd5a:kaede*-labeled chondrocytes eventually co-express *col10a1a:Citrine*, which is the earliest known marker for HCs (Girkontaite et al., 1996). In tetrapod GPs, a pre-hypertrophic zone between the PZ and the HZ is defined by expression of *Col10a1* and high levels of *Sox9*, in contrast to the mature HZ, which completely lacks *Sox9* expression. *Sox9* functions in these pre-hypertrophic cells to block further hypertrophic differentiation (Akiyama et al., 2004; Bi et al., 2001; Hattori et al., 2010; Zhao et al., 1997). In contrast to previous studies in which HZ zone markers such as *ihha*, *ihhb* and *runx2b* were shown to be expressed in the middle of the ch cartilage starting at 120 hpf (Eames et al., 2010, 2011; Felber et al., 2011), our study shows that *entpd5a:kaede* is co-expressed with *ihha* in the developing HZ of the ch cartilage 2 days earlier, at 72 hpf. In addition, the expression of *entpd5a:kaede* in zebrafish chondrocytes is sensitive to CyA treatments that perturb Hh signaling, which at later stages in tetrapods is required for proliferating chondrocytes to embark on hypertrophic differentiation as well as subsequent ossification (Chung et al., 2001; Mak et al., 2008; St-Jacques et al., 1999), further supporting our observations.

*entpd5a* is better known for its expression in bone, including osteoblasts surrounding the notochord that later contribute to the vertebrae. Osteoblast differentiation requires the transcription factor Sp7 (also known as Osterix) (Nakashima et al., 2002), but some osteoblasts, such as those surrounding the notochord that express *entpd5a*, are *sp7* negative (Huitema et al., 2012; Lleras Forero et al., 2018; Wopat et al., 2018; Yu et al., 2017). A subset of chondrocytes within the ch cartilage also expresses *sp7* and contributes to matrix mineralization starting at 120 hpf, although how this cell population relates to *entpd5a^+^* chondrocytes is unclear (Hammond and Schulte-Merker, 2009). A subset of osteoblasts can be traced to *col2a1a*-expressing chondrocytes in juvenile zebrafish cartilage GPs, suggesting that some HCs differentiate into osteoblasts (Giovannone et al., 2019), possibly including those that express *entpd5a*. Given that not all *col10a1a:Citrine*^+^ chondrocytes co-expressed *entpd5a:kaede*, it is possible that *entpd5a* may be an early marker for chondrocytes that will later trans-differentiate into osteoblasts.

### Pthlh signaling controls spatial-temporal patterns of embryonic cartilage hypertrophy

We have shown that *pthlha* is expressed at the dorsal and ventral edges of the ch cartilage condensation and other pharyngeal arches at 48 hpf, 24 h prior to cartilage differentiation and detection of the first cells expressing *entpd5a:kaede* in the pre-HZ. Loss of *pthlha* leads to dorsal expansion of the *entpd5a:kaede*-expressing cell population. Conversely, ectopic *pthlha* expression in the ch cartilage can disrupt *entpd5a* expression and delay ossification, depending on the number and location of *pthlha*-expressing cells. In some cases, ectopic *pthlha* eliminates *entpd5a:kaede*-expressing cells, whereas in others it induces ectopic *entpd5a:kaede* expression, presumably depending on other factors, including *pthlha* autoregulation. *Pthlh* mRNA levels appear to be kept relatively low via a translation feedback mechanism (Broadus et al., 2007; Chen et al., 2007). Together, these results suggest that all chondrocytes in the ch cartilage have the potential to undergo hypertrophy, depending on where they are with respect to the Pthlha gradient. Presumably, Pthlha protein, secreted from the ends of the skeletal condensation, restricts HZ to the center and controls the onset of hypertrophy. Similarly, at later stages, loss-of-function mutations in murine *Pthlh* lead to increased and premature hypertrophic cartilage differentiation, reduced proliferation, and lethality associated with abnormal skeletal morphology (Karaplis et al., 1994). *Pthlh* represses *Runx2* transcription as well as *Ihh* expression, both of which normally promote hypertrophy (Li et al., 2004; Yoshida et al., 2004). Upregulation of Pthlh signaling in long bones in mice, either via overexpression of Pthlh or constitutively active Pthr overexpression, delays ossification but does not appear to alter HZ position (Schipani et al., 1997; Weir et al., 1996). This difference may reflect the fact that zebrafish cartilages have orders of magnitude fewer cells than mammalian long bones, such that a few ectopic Pthlh-expressing cells can have profound effects on cartilage pattern.

Surprisingly, the *entpd5a:kaede*-labeled chondrocytes that mark the pre-HZ expand dorsally but not ventrally in *pthlha* F0 mutants. Furthermore, ectopic *pthlha* expression can result in *entpd5a:kaede*-expressing chondrocytes on the ventral side. The causes of these dorsal-ventral asymmetries remain unclear. One possibility is the additional influence of other related ligands and receptors. One such signal could be *pthlhb*, which is expressed in some craniofacial cartilages and bones but apparently not in ch cartilage (Yan et al., 2012). However, we detect no *pthlhb* expression in larval ch cartilage at the stages we have examined. In addition, zebrafish have two Pthlh receptors, Pth1ra and Pth1rb, the activation sensitivity of which depends on the ligand (Rubin and Jüppner, 1999). Although *pth1ra* expression appears to be uniform, *pth1rb* may play a role in this asymmetry. Moreover, Parathyroid Hormone 1 (Pth1) acts like Pthlh in cartilage and can be carried in the bloodstream in rodents (Jee et al., 2018; Yukata et al., 2018). Finally, *pth4* is an ancestral Pth found in zebrafish but lost in eutherian mammals, that is expressed in the central nervous system, activates Pth1r receptors, and represses mineralization in the skeleton at long range (Suarez-Bregua et al., 2017). One or more of these factors may play a role in spatiotemporal patterns of cartilage hypertrophy, which warrants further investigation.

We also find that *pthlha* mutants have decreased cartilage proliferation at 120 hpf, suggesting that the role for *Pthlh* in maintaining proliferative chondrocytes is conserved. Cartilages are smaller in *Pthlh* mutant mice due to reduced proliferation (Karaplis et al., 1994). Increased hypertrophic differentiation in HZs upregulates *Runx2* and *Fgf18* in the perichondrium, which inhibits proliferation in the PZ, thereby limiting the number of proliferating chondrocytes that transition into the HZ (Hinoi et al., 2006; Liu et al., 2002; Ohbayashi et al., 2002).

### Mechanical force promotes cartilage hypertrophy independently of proliferation

Our analyses of paralyzed zebrafish suggest that mechanical force promotes formation of the pre-HZ in embryonic cartilages. Paralysis induced by α-BTX injection disrupts localization of *pthlha* and reduces *ihha* expression in the ch cartilage, as well as preventing later *entpd5a* expression and subsequent ossification. These results appear to be consistent with previous studies (Nowlan et al., 2008; Saitoh et al., 2000; Wang and Mao, 2002). However, this occurs at stages prior to 96 hpf when there is no proliferation in zebrafish, suggesting that the effects of mechanical force on hypertrophic differentiation and bone growth are due to direct effects on the chondrocyte differentiation program and not indirectly through the rate at which chondrocytes move away from the Pthlh source due to changes in proliferation. Paralysis reduces proliferation in the ventral ch cartilage at later stages, consistent with previous studies in other vertebrates (Germiller and Goldstein, 1997; Wang and Mao, 2002).

Our results suggest that the effects of mechanical force on chondrocytes are secondary to changes in Pthlh and Ihh expression. Mechanical force has been shown to promote *Pthlh* and *Ihh* expression both in mandibular chondrocytes as well as in fibrocartilage at developing entheses (Chen et al., 2007; Jahan et al., 2014; Rais et al., 2015). Chondrocytes may also sense force cell-autonomously because they express mechanosensitive channels, including *Piezo1* and *Piezo2* (Lee et al., 2014; Servin-Vences et al., 2017; Wu et al., 2017) as well as *Trpv4* (Muramatsu et al., 2007; Nilius et al., 2004; O'Conor et al., 2014; Servin-Vences et al., 2017). *Trpv4* activity is required for *Sox9* expression in differentiating chondrocytes *in vitro* (Muramatsu et al., 2007) and promotes the production of type II collagen and other ECM proteins in differentiating chondrocytes (O'Conor et al., 2014).

### Distinct temporal deployment of the Pthlh/Ihh feedback loop in different skeletal growth zones

Most studies of endochondral development and function have focused on epiphyseal GPs of tetrapod limb bones. Here, it is thought that the Pthlh/Ihh feedback loop is not active until later (e.g. 3 weeks post-hatching in chick GPs), because *Pth1r* expression is not detected in PCs and HCs before this stage (Vortkamp et al., 1996). However, endochondral bones come in many different shapes, reflecting distinct developmental regulatory mechanisms, such as the mirror-image organization of cranial synchondroses or GZs that produce bidirectional growth as well as a greater reliance on proliferation versus hypertrophy in teleost GPs and GZs (Heubel et al., 2021). In addition, many cranial cartilages and bones, including the ch, arise from neural crest rather than mesoderm, which forms the limb skeleton, and these distinct embryonic origins of endochondral bones may also underlie distinct regulatory mechanisms involved in GP and GZ establishment.

Our results suggest that, in contrast to tetrapod GPs, Pthlh signaling actively patterns the GP of the ch cartilage in zebrafish much earlier, as chondrocytes differentiate in the embryo. Zebrafish *pth1r* is ubiquitously expressed throughout embryonic development (Gray et al., 2013), we detect expression of *pth1ra* in the developing ch (data not shown), and knockdown of Pth1r expression disrupts craniofacial cartilage morphology (Kwong and Perry, 2015). *pthlha* depletion in zebrafish also leads to increased endochondral bone mineralization in the larval craniofacial skeleton, consistent with its roles in skeletal development in mammals but at a much earlier stage (Yan et al., 2012). Together, our data provide an example of a negative-feedback loop involved in spatial patterning of cartilage that is initiated by the repressive signal (Pthlh), instead of the activator signal (Ihh), suggesting that a similar sequence of events occurs during GP formation in other skeletal elements. This may also be a common feature of the establishment of negative-feedback loops in a variety of other tissue patterning systems.

## MATERIALS AND METHODS

### Ethics statement

All animals were handled in accordance with good animal practice as defined by the relevant national and/or local animal welfare bodies, and all animal work was approved by the University of California, Irvine Institutional Animal Care and Use Committee.

### Animals and transgenics

Zebrafish (*Danio rerio*) of the AB strain were raised and staged as previously described (Kimmel et al., 1995; Schilling and Kimmel, 1997). Equal numbers of males and females were used for breeding, typically 1-2 years of age. Embryos and larvae were anesthetized in tricaine (MS-222) (Westerfield, 2000). *Tg(sox10:lyn-tdTomato)* ir1040 was previously generated in our lab (Schilling et al., 2010). Transgenic lines *TgBAC(entpd5a:Kaede) hu6867*, *TgBAC(entpd5a:KillerRed) hu7478* and *TgBAC(col10a1a:Citrine) hu7050* were kindly provided by Dr Stefan Schulte-Merker (Lleras Forero et al., 2018; Mitchell et al., 2013).

Constructs for mosaic *pthlha* expression were generated using the Gateway Tol2 system (Tol2Kit; Kawakami and Shima, 1999; Kwan et al., 2007) in combination with Gibson cloning (Gibson et al., 2009). Primer pairs (upper case designates primer sequences that are complementary to the target sequence in the PCR reaction; lower case designates overhanging primer sequences used for further cloning in Gibson reactions): caaaaaagcaggctggacATGAGGATGTTGTGTTGCAG and ccggatccGCAGCTGTACGGCTGCAG were used to amplify the open reading frame of *pthlha* from the pGEMT-*pthlha* by PCR. Primer pairs: acagctgcGGATCCGGAGCCACGAAC and gtgctatagggctgcaTCAGGAGAGCACACACTTGC were used to amplify *T2A-eGFP-CAAX* from the Tol2Kit plasmid p3E-*2A-EGFP-CAAXpA* (plasmid #458) by PCR. Primer pairs tgagagagGGATCCGGAGCCACGAAC and gtgctatagggctgcaCTTGTACAGCTCGTCCATGC were used to amplify *T2A-nlsmCherry* from the Tol2Kit plasmid p3E-*2A-nlsmCherrypA* (plasmid #766) by PCR. To generate the pME-*pthlha-T2A-eGFP-CAAX* middle entry vector, a NcoI/XbaI-digested Tol2Kit pME-*eGFP* vector (plasmid #455) was combined with *pthlha* and a *T2A-eGFP-CAAX* amplicon in a Gibson reaction. Similarly, a pME-*pthlha-T2A-nlsmCherry* middle entry vector was generated using *pthlha* and *T2A-nlsmCherry* amplicons by Gibson cloning. A *col2a1a* promoter was used from the p5E-*col2a1a* plasmid (Dale and Topczewski, 2011). pDestTol2pA2-*col2a1a:pthlha-T2A-eGFP-CAAX* and pDestTol2pA2-*col2a1a*:pthlha-T2A-nlsmCherry were assembled according to the Tol2Kit protocol. All plasmids were transformed into DH5α-competent cells (in-house generated; Inoue et al., 1990). Transposase mRNA was synthesized from the pCS2FA-transposase plasmid (Kwan et al., 2007), linearized with NotI, using Invitrogen mMESSAGE mMACHINE™ T7 ULTRA Transcription Kit (AM1345). 500 pl of cocktail mixes containing 40 ng/μl of plasmid and 60 ng/μl of transposase mRNA were injected into one-cell-stage embryos.

### Alizarin Red staining

Alizarin Red S (EM Science, AX0485-3) staining was carried out as previously described with some modifications (Walker and Kimmel, 2007). Briefly, Alizarin Red was dissolved to 0.5% in H_2_O as a stock solution. Staining solution was prepared by adding 10 μl of this stock to 1 ml of embryo medium (EM; Westerfield, 2000) without Methylene Blue. Live embryos were kept in staining solution at 28.5°C for 1 h. After removing the staining solution and rinsing embryos three times, embryos were left in EM for 30 min prior to live imaging.

### α-BTX injections

Embryo paralysis was achieved by injecting 5 nl of a 500 μM solution of α-BTX (Tocris, 2133) into the bloodstream near the heart outflow just posterior to the otic vesicle of 68 hpf as described previously (Subramanian et al., 2018).

### BrdU labeling and staining

BrdU (Sigma-Aldrich, B9285-250MG) labeling and staining was carried out as previously described with some modifications (Kimmel et al., 1998). Primary mouse monoclonal anti-BrdU (Clone BU 33) antibody (Sigma-Aldrich, B2531) was used at a 1/100 dilution. Secondary Alexa-647 Donkey anti-mouse IgG (H+L) antibody (Jackson ImmunoResearch Laboratories, 715-607-003) was used at 1/200.

### CyA treatment

A stock solution of CyA (LC Labs, C-8700) was prepared by resuspension in 100% ethanol to a final concentration of 10 mM. This solution was aliquoted and stored at −20°C. Zebrafish embryos were treated at 72 hpf with CyA diluted to a final concentration of 50 μM and an ethanol concentration of 0.5% in EM without Methylene Blue. A solution of 0.5% ethanol in EM was used to treat controls. Treatments were carried out in a 28.5°C incubator. Embryos were removed from CyA at 96 hpf, washed with EM five times, placed in EM and raised to 120 hpf for live imaging.

### CRISPR/Cas9 mutagenesis

Multiplex CRISPR/Cas9 mutagenesis was performed to generate F0 *pthlha* mutants (Wu et al., 2018). Spacers used to make *pthlha* primers for guide RNA synthesis: GGGCATCGACGGGCCGGCCG, AGGATTTTAAGCGGCGCATG, TCCGGGAGGCGCAGCAGCCC and TGGTGCCGCCGGCGGGTTTG. After assembling pooled guide-RNA (gRNA) templates by PCR, gRNAs were synthesized using the MEGAshortscript T7 Transcription kit from Invitrogen (AM1354). Alt-R S.p. Cas9 nuclease 3NLS protein was obtained from Integrated DNA Technologies (1074182). An injection mix was prepared by diluting the Alt-R S.p. Cas9 nuclease 3NLS protein to 5 μM and pooled *pthlha* gRNAs to approximately 800 ng/μl in H_2_O. This mix was incubated at 37°C for 5 min, then injected at approximately 500 pl/embryo at the one-cell stage.

### Testing multiplex *pthlha* gRNAs

Multiplexed *pthlha* gRNAs and Cas9 protein were co-injected into one-cell-stage wild-type embryos as previously described. At 24 hpf, 14 injected and 14 uninjected embryos were each placed into PCR tubes containing 35 μl of 50 mM NaOH. Genomic DNA was extracted by incubation at 98°C for 20 min and then neutralized with the addition of 5 μl of 1 M Tris pH 8.0. Each extracted genomic DNA sample was used as template in four PCR reactions to amplify each target site (see Table S1 for primer sequences). PCR products were then run on 10% native PAGE gels and stained with GelStar Nucleic Acid Gel Stain (Lonza, 50535) (Ota et al., 2013).

### Imaging

Embryos for live imaging were embedded in 1% low melting point agarose (Apex, 9012-36-6) diluted in EM containing tricaine. Embryos labeled by *in situ* hybridization and hybridization chain reaction (HCR) were mounted on slides, then imaged on a Zeiss Axioplan 2 microscope equipped with a MicroPublisher 5.0 RTV camera using Volocity software (Improvision) and on a Leica Sp8 confocal microscope equipped with a HC PL APO CS2 40x/1.10 W objective, respectively. All live imaging was carried out using either a Leica Sp8 confocal microscope equipped with a HC PL APO CS2 40×/1.10 W objective, or a Nikon ECLIPSE Ti confocal microscope equipped with a Plan Apo VC 20×/0.75 DIC N2 objective. ImageJ/Fiji was used for image processing. R-suite and plugins dplyr, ggplot2, ggsignif, and plyr were used for quantification, analysis and statistical tests (Welch two-tailed *t*-test and Wilcoxon test).

### *In situ* hybridization

To make a *pthlha* probe, primers CAGGACGTAATGCTGAGCCG and GTGGACGTGAGCATTTAGGC were used to amplify *pthlha* cDNA prepared from 72 hpf embryo mRNA. The PCR product was cloned into Promega's pGEM-T Easy Vector (A1360) to make a pGEMT-*pthlha* plasmid and transformed into DH5α cells. The plasmid was digested with NcoI and probe synthesized using Roche DIG RNA Labeling Mix (11277073910) and SP6 RNA Polymerase (10810274001). The *ihha* probe has been described previously (Avaron et al., 2006). Whole-mount *in situ* hybridization was carried out as previously described with some modifications (Thisse et al., 1993). Synthesized probe was diluted in hybridization buffer to 100 pg/μl. Anti-Digoxigenin-AP antibody Fab fragments (Roche, 11093274910) were used at a dilution of 1/1000. *In situ* HCR (Choi et al., 2014) was performed according to the HCR v3.0 protocol with probes and amplifier labels ordered from Molecular Instruments. Probes and amplifier labels used were as follows, *pthlha* (NM_001024627.2; 17-probe set) and *pthlhb* (NM_001043324.1; 9-probe set) in B3 with B3 Alexa Fluor 647, *ihha* (NM_001034993.2; 20-probe set) and *ihhb* (NM_131088.1; 20-probe set) in B2 with B2 Alexa Fluor 488, and *sox9a* (NM_131643; 17 probe set) in B1 with B1 Alexa Fluor 546.

## Supplementary Material

Supplementary information

Reviewer comments
