# Supplementary material for: Pthlha and mechanical force control early patterning of growth zones in the zebrafish craniofacial skeleton
Source: Development. 2022 Jan 20;149(2):dev199826. doi: 10.1242/dev.199826 (PMC8917414; doi:10.1242/dev.199826)
Supplement: Supplementary information [file develop-149-199826-s1.pdf]

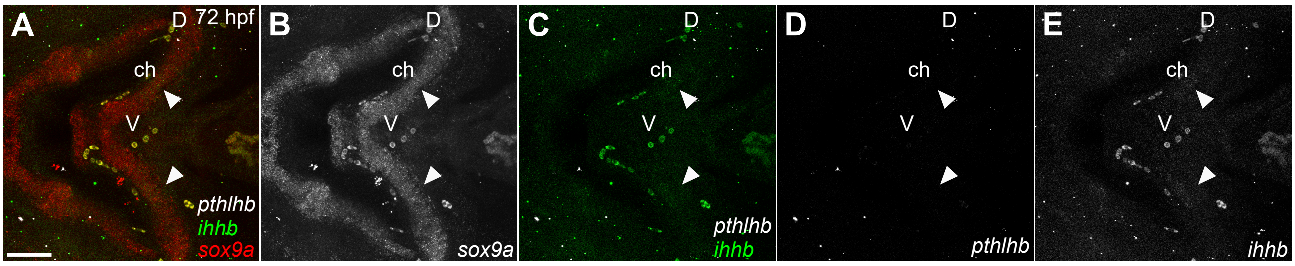

Fig. S1. *pthlhb* and *ihhb* expression at 72 hpf

HCR *in situ* hybridization for *pthlhb*, *ihhb*, and *sox9a* in wild-type embryos at 72 hpf (A-E). White arrowheads indicate the ceratohyal *ihhb* expression zones. All micrographs are ventral view Z-projections with anterior to the left. ch=ceratohyal, D=dorsal, V=ventral. Scale bar: 50 μm (A-E).

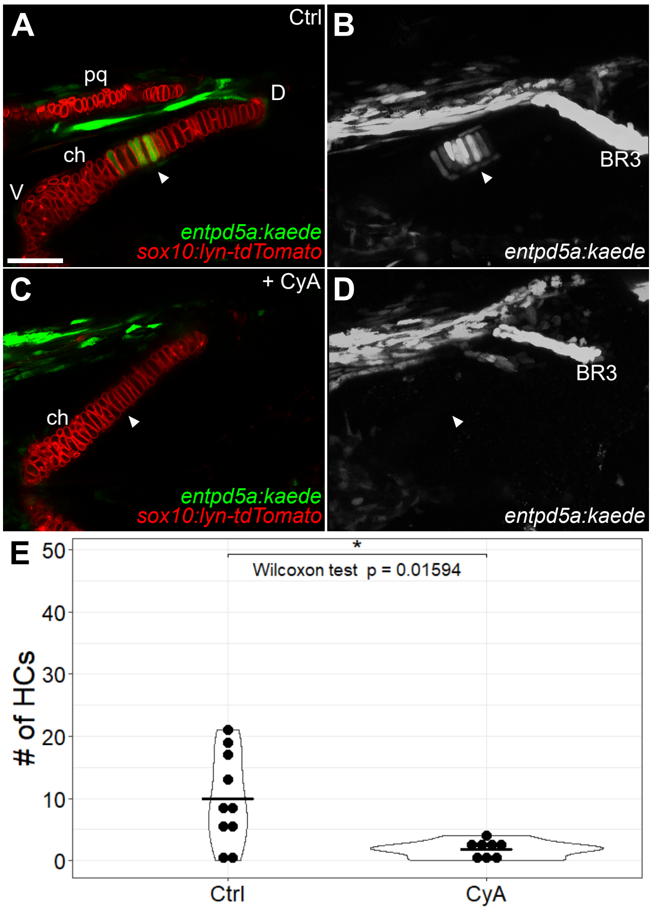

Fig. S2. Inhibition of Hh signaling reduces numbers of *entpd5a*-labeled ceratohyal chondrocytes

(A-D) Cyclopamine (CyA) treatments at 68 hpf of *sox10:lyn-tdTomato;entpd5a:kaede* double transgenic embryos, imaged at 120 hpf. (A, B) Vehicle-treated control (Ctrl) embryos. (C, D) CyA-treated embryos. (E) Quantification of *entpd5a:kaede*-labeled chondrocyte numbers. White arrowheads indicate the position of the hypertrophic zone. (A, C) Optical slices. (B, D) Z-projections. pq= palatoquadrate, ch=ceratohyal, D=dorsal, V=ventral, BR3=branchiostegal ray 3. Scale bar: 50 µm (A-D).

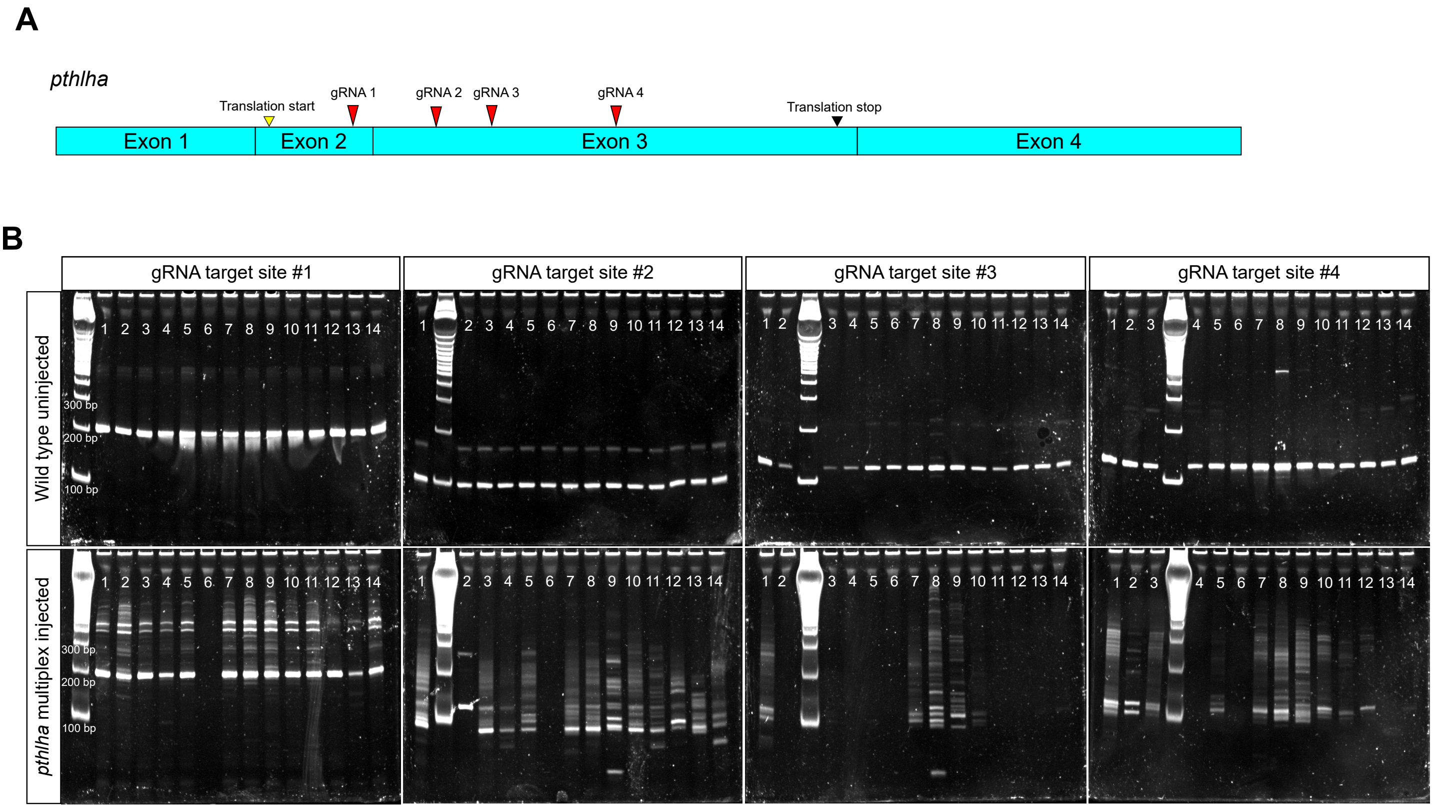

Fig. S3. *pthlha* multiplex gRNA target sites

A) Diagram of the zebrafish *pthlha* transcript depicting the four sites targeted (red triangles) as well as the translational start and stop sites (yellow and black triangles, respectively). (B) Heteroduplex Mobility shift Assay (HMA; Ota et al. 2013) illustrating cutting of each gRNA at their respective target sites in 14 unique 24 hpf embryos that were injected with multiplexed *pthlha* gRNAs and Cas9 protein at the one-cell stage and 14 uninjected control embryos. Cutting of the targeted genomic region by CRISPR-Cas9 results in unique insertion and deletion events. When the target region is amplified by PCR, heteroduplexes form between mutant and wild type DNAs. These secondary structures have a laddering pattern when analyzed by native PAGE. Each gel lane represents a single embryo assayed at each target site. Some non-specific amplification products were for gRNA 2 and gRNA 4 amplicons.

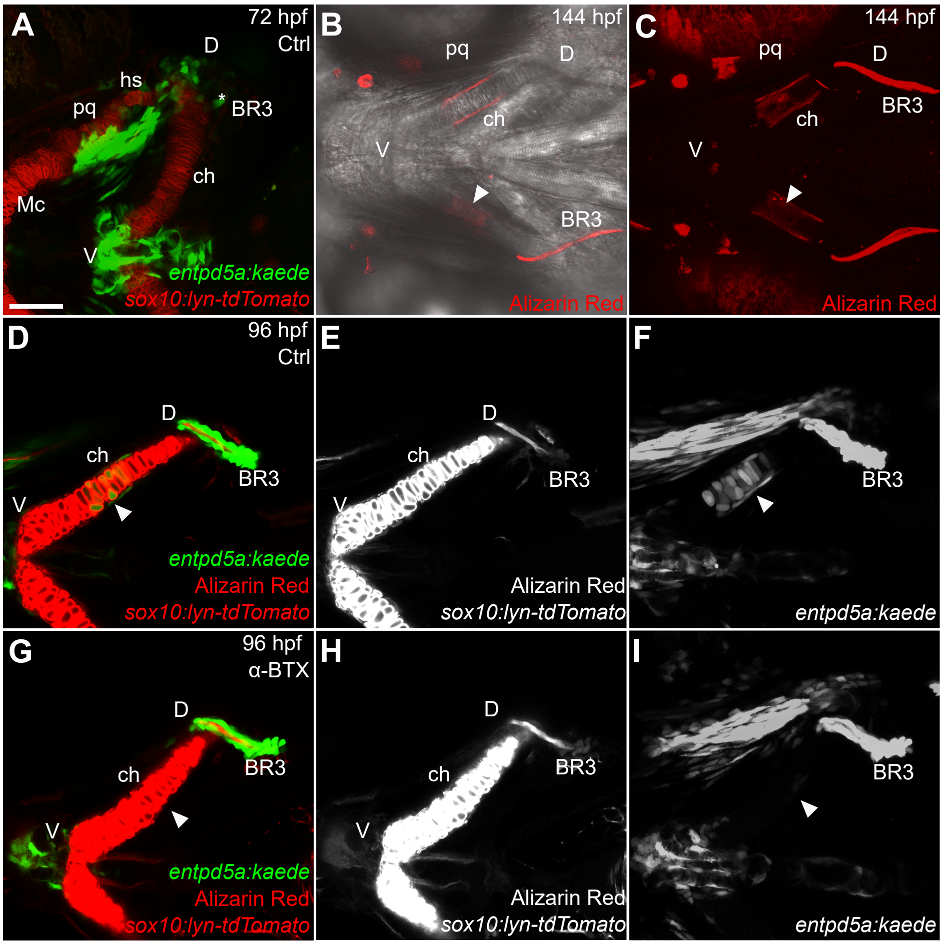

Fig. S4. HZ loss during paralysis is not due to developmental delay.

(A) Live imaging of branchiostegal ray 3 (BR3) development in a wild-type *sox10:lyn-tdTomato;entpd5a:kaede* double transgenic embryo at 72 hpf, when BR3 has not yet formed. (B, C) Live imaging of BR3 development in an Alizarin red-stained wild-type embryo at 144 hpf, when the BR3 has already formed. (B) An optical slice showing the DIC channel. (D-I) Live imaging of a wild-type control (D-F) and α-BTX-injected paralyzed (G-I) *sox10:lyn-tdTomato;entpd5a:kaede* embryos stained with Alizarin Red at 96 hpf. BR3 develops normally and stains with Alizarin Red regardless of paralysis. (B, D, E, G, H) Optical slices. (A, C, F, I) Z-projections. White arrowheads indicate the position of the hypertrophic zone, which is absent in paralyzed embryos. pq=palatoquadrate, ch=ceratohyal, hs=hyosymplectic, Mc=Meckels, D=dorsal, V=ventral All micrographs are ventral view optical slices with anterior to the left. Scale bar: 50 µm (A-I).

Table S1. Primers used to assay *pthlha* multiplex gRNAs

Primer	Sequence	Description
pthlha_gRNA1_F	GAGGATGTTGTGTTGCAGAC	Flanks gRNA1 site. 196 bp
pthlha_gRNA1_R	GATCAGCAGCTCTAAGAGCA	Flanks gRNA1 site. 196 bp
pthlha_gRNA2_F	GAAGCGTTCAGTGACCCA	Flanks gRNA2 site. 102 bp
pthlha_gRNA2_R	TGCACTTCATGCAGGAGC	Flanks gRNA2 site. 102 bp
pthlha_gRNA3_F	GCTCCTGCATGAAGTGCA	Flanks gRNA3 site. 123 bp
pthlha_gRNA3_R	CCTGCAGGCAGTGTGATG	Flanks gRNA3 site. 123 bp
pthlha_gRNA4_F	CATCACACTGCCTGCAGG	Flanks gRNA4 site. 122 bp
pthlha_gRNA4_R	GGTTTGTGCCCTCCTCATC	Flanks gRNA4 site. 122 bp
