# Supplementary material for: Pthlha and mechanical force control early patterning of growth zones in the zebrafish craniofacial skeleton
Source: Development. 2022 Jan 20;149(2):dev199826. doi: 10.1242/dev.199826 (PMC8917414; doi:10.1242/dev.199826)
Supplement: Reviewer comments [file develop-149-199826_review_history.pdf]

Pthlha and mechanical force control early patterning of growth zones in the zebrafish craniofacial skeleton

Diego J. Hoyle, Daniel B. Dranow and Thomas F. Schilling
DOI: 10.1242/dev.199826

Editor: Steve Wilson

Review timeline

Original submission:	24 May 2021
Editorial decision:	30 June 2021
First revision received:	2 November 2021
Editorial decision:	2 December 2021
Second revision received:	3 December 2021
Accepted:	7 December 2021

Original submission

First decision letter

MS ID#: DEVELOP/2021/199826

MS TITLE: Pthlha and mechanical force control early patterning of growth zones in the zebrafish craniofacial skeleton

AUTHORS: Thomas F Schilling, Diego J Hoyle, and Daniel Dranow

I hope all is well. I have now received all the referees' reports on the above manuscript, and have reached a decision. The referees' comments are appended below, or you can access them online: please go to BenchPress and click on the 'Manuscripts with Decisions' queue in the Author Area.

As you will see, the referees like your manuscript but have some criticisms and suggestions for improvements. If you are able to revise the manuscript along the lines suggested, I will be happy receive a revised version of the manuscript. Please also note that Development will normally permit only one round of major revision.

We are aware that you may be experiencing disruption to the normal running of your lab that make experimental revisions challenging. If it would be helpful, we encourage you to contact us to discuss your revision in greater detail. Please send us a point-by-point response indicating where you are able to address concerns raised (either experimentally or by changes to the text) and where you will not be able to do so within the normal timeframe of a revision. We will then provide further guidance. Please also note that we are happy to extend revision timeframes as necessary.

Please attend to all of the reviewers' comments and ensure that you clearly highlight all changes made in the revised manuscript. Please avoid using 'Tracked changes' in Word files as these are lost in PDF conversion. I should be grateful if you would also provide a point-by-point response detailing how you have dealt with the points raised by the reviewers in the 'Response to Reviewers' box. If you do not agree with any of their criticisms or suggestions please explain clearly why this is so.

© 2022. Published by The Company of Biologists under the terms of the Creative Commons Attribution License (https://creativecommons.org/licenses/by/4.0/).

Reviewer 1

Advance summary and potential significance to field

This exciting study by Hoyle et al. reveals that pthlha signaling and muscle force initiate zonal patterning of the growth plate by specifying pre-hypertrophic chondrocytes within a delimited domain in the cartilage anlage of the ceratohyal (ch) bone in the zebrafish jaw. Novel aspects of this study include the 1) use of entpd5a expression as a novel marker for early pre-hypertrophic chondrocytes and 2) discovery of factors that establish the pthlh-ihh negative feedback loop, which is a key regulator of bone length in development and disease.

Comments for the author

While this is a clearly written and elegant study, there are two aspects that could be strengthened: 1) a more complete expression analysis of pthlha and its receptors in the developing ch is needed (see notes 2 and 3 under General feedback) and 2) tests to determine the extent to which mechanical force and Pthlh signaling function separately or are linked during growth plate patterning should be done (see note 6 under General feedback).
General feedback:
Introduction:
1. Provide information about entpd5a. What is the function of the gene and why was it looked at? Is it just a marker for pre-hypertrophic chondrocytes or is predicted that its role in phosphate/pyrophosphate metabolism has a functional role in chondrocyte hypertrophy?

Results:
2. In Figure 2, it is very difficult to appreciate that the localization of pthlha expression is restricted to the dorsal and ventral ends of the ch cartilage. Since this is the foundation of the model, consider different images and/or the use quantitative measures. Another option is to use RNAscope to enhance resolution.
3. It is proposed that initiation of cartilage hypertrophy is both spatially and temporally regulated by local exposure to pthlh signaling. However, it is unclear what cells the ligand is signaling to. Zebrafish have two receptors:
Pthr1a and Pthr1b. In the discussion it is said that pthr1 is expressed ubiquitously throughout embryogenesis (Gray et al ., 2013). This is based on qPCR and in situ experiments between 1-5 dpf that do not distinguish between the two isoforms. The expression pattern of the receptor should be revisited at the stages being studied here using in situ probes that distinguish between the isoforms. This could resolve how pthlh mechanistically acts at a distance in the developing growth plate.
4. It is said that ihha/b are not expressed, but that Hh signaling is required for expression of the entpda transgene. The authors then say that shh from the endoderm serves as the likely source of Hh. Proximity of shh expression to the cells of interest should be shown to support this idea.
5. On page 10, it says that CRISPR-induced deletions of pthlha were confirmed in injected embryos by detection of early ossification in entpda+ bone. However later it is said that BR3, which is an entpda+ bone, was indistinguishable from control. This should be clarified.
6. Following paralysis, entpda+ pre-hypertrophic chondrocytes in the middle of the ch are lost and, later, proliferating chondrocyte at the ventral region of the ch are significantly reduced. How does this connect with pthlh and Hh signaling and, more specifically, with expression of pthlha, its receptors, and shh in the endoderm? Is the loss of entpda+ cells and decreased cell proliferation secondary to loss of force-regulated pthlh or shh expression?
Expression analysis following paralysis would help connect mechanical force and cell signaling in this study.
7. The proliferative zones at the dorsal and ventral tips of the ch seem to form at the interface between pthlh+ cells and pre-hypertrophic/hypertrophic cells. Is this the case? If so, is it then expected that pthlh misexpression would lead to aberrant localization of the proliferative zones after 120 hpf?
This would further support the idea that pthlh signaling regulates patterning of the growth plate zones.
Discussion:
8. In Figure 1 I-L , it is shown that not all entpda+ cells are col10+ in the ch cartilage at 144 hpf. This should be further discussed on page 18, second paragraph. Is it thought that there are there

© 2022. Published by The Company of Biologists under the terms of the Creative Commons Attribution License (https://creativecommons.org/licenses/by/4.0/).

several populations of hypertrophic chondrocytes (entpda+ , col10+, entpda+/col10+)? Or is it believed that entpda+ cells become entpda-/col10+? Are entpda+ hypertrophic chondrocytes thought to be a subpopulation that undergoes trans-differentiation into osteoblasts since entpd5a is traditionally a marker for osteoblasts and involved in mineralization?

Specific feedback:
1. Introduction, page 3, second paragraph: It is states that hypertrophic chondrocytes within the growth plate undergo apoptosis. While this has been the dogma for many years, there is now evidence that at least some hypertrophic chondrocytes survive and transdifferentiate into osteoblasts/osteocytes (Yang et al., 2014, PNAS; Yang et al., 2014, Cell Res; Kobayashi et al., 2014, Nature; Jing et al., 2015, JDR; Park et al., 2015, Biology Open)
2. Introduction, second paragraph: The sentence that begins with “This prevents…” should end with a period (delete the comma).
3. In Figure 2 legend, please include a statement that entpd5a:killer red+ cells were not found at 48 hpf (similar to what is said in the results section).
4. Supplemental Figure 1 legend: “HZ” is not shown in the figure and should be deleted.
5. Figure 3D-E: There are two white arrowheads. While one clearly points to the HZ, it is not clear what the other indicates from the Figure legend.
6. Figure 3 legend: Remove the second comma between “ch= ceratohyal” and “D =dorsal”.
7. Figure 4 legend: “HZ” is not shown in the figure and should be deleted.
8. Discussion, page 22: The paragraph that starts with “Chondrocytes may sense force…” is repetitive with the introduction.
9. Discussion, page 23: To what extent does the embryonic origins of endochondral bone (neural crest versus mesoderm) come into play with respect to distinct regulatory mechanisms?

Reviewer 2

Advance summary and potential significance to field

The paper presents a detailed analysis of the role of Pthlha in early patterning of developing cartilages in the zebrafish ceratohyal. The authors identify entpd5a as a marker for the pre-hypertrophic zone (pre-HZ) and show that expression in cartilage cells is depended on pthlha and hedgehog signaling. In addition, they show that the formation of the pre-HZ is dependent on mechanical forces. While a role for Pthlh and mechanical forces in cartilage development has been shown before, the present study for the first time shows the role during the early stages of cartilage patterning.

Comments for the author

The paper is well written, and conclusions are supported by the presented data.
However, the manuscript could be improved through the following additional experiments:
1. Essential addition While the authors do show the expected phenotypic change after CRISPR-Cas9 injection, no successful targeting of the pthlha locus is shown. Since the guides have not been used before, a T7 assay or similar control should be added to show guide efficiency and presence of CRISPR induced cuts.
2. Potential experimental additions
The analysis of the expression of the b orthologues of ihh and pthlh would complete the analysis of the role of ihh and pthlh in early cartilage patterning.
While the authors do describe ihh as a potentially important gene in the establishment of the expression of entpd5 in the pre-HZ, only the expression of ihha is analyzed. Similarly, the expression of pthlhb in early ceratohyal developmental stages has not been analyzed before.
Minor non-experimental changes and additions:
- Please add which HCR label was used
- The addition of a schematic indicating the guide target in the pthlha gene locus would help to clarify the design of the experiment.
- A closeup of the ch in figure 2C-E would make it easier to see the pthlha expression in the ch

© 2022. Published by The Company of Biologists under the terms of the Creative Commons Attribution License (https://creativecommons.org/licenses/by/4.0/).

Reviewer 3

Advance summary and potential significance to field

Hoyle, Dranow and Schilling describe the patterning of the growth zone of the ceratohyal cartilage of zebrafish. They demonstrate that Pthlha is expressed early in the chondrocyte precursors, becoming spatially restricted. They show that loss of Pthlha leads to expansion of the entpd5a expression (previously more closely linked to mineralization of bone). They also demonstrate that paralysis leads to reduction of entpd5 expression and subsequent ossification.

While not much of the work is completely novel e.g. the expression of pthlha has been previously characterized and it's down regulation (by MO) shown to lead to premature ossification of the ceratohyal.
Paralysis has also been shown to regulate migration, intercalation and size of chondrocytes in zebrafish paralysis is known to reduce hypertrophy in other models (chick/mouse) and the entpd5a transgenic has been previously described. The paper does put the elements of the story together in a helpful way that makes comparison to mammalian chondrogenesis clearer, and uses some elegant mosaic experiments to study local effects of pthlha misimpression on ossification.

Comments for the author

The paper is well written and easy to follow. The imaging is high quality and the figures are well presented.
In general I have no suggestions for the early sections of the paper which are logical and well done. That said I think for completeness it would have been good to see what happens in the mosaic expressing zebrafish later in development, i.e. does early misimpression of pthlha lead to continued local disruption to the HZ/ossification with consequences for the patterning of the element in late larval/juvenile fish or is this effect transient?
The paralysis section feels the most preliminary. As the relationship to mechanical force is a selling point for the paper, and indeed features in the title it feels as though this section would be improved by inclusion of more data for at the very least comparison t other published studies. While the authors state 'previous studies have shown mechanical forces influence GP dynamics but effects largely interpreted as indirect due to changes in proliferation' I'm not sure this is the case. Work in chick and mouse such as from Paula Murphy and Niamh Nowlan's group have looked at hypertrophy and ossification of chick long bones in relation to paralysis using FE and also in situs for ColX etc (e.g. Nowlan, Murphy Prendergast J Biomech 2008, Nowlan, Prendergast, Murphy Plos Comp Biol 2008), and other groups too.
The paralysis section I think would benefit from comparison with and referencing of other work in which effects of paralysis on cartilage have been studied in fish, including Schwarz and Zelzer's Dev Biol 2012 paper where they showed defects in intercalation and hypertrophy (though they didn't use word hypertrophy
- calling it 'stacking' the outlines in their figures show same effects clearly) following MS22 treatment and use of the Nic mutant. There are multiple options the authors could use to follow up and strengthen the mechanical forces section. They could use other drugs (to test whether flaccid vs rigid paralysis have different effects - e..g ones used previously in zebrafish Brunt et al 2016 Osteoathrtis cartilage), could consider FE modeling (e..g Brunt et al J Biomech 2015) or following up with antibodies or in situs for Piezo1/2 Trpv4 (which they themselves mention in the discussion) or looking into Yap/Taz signaling. Ideally one would show the relationship of mechanosensitivity to entpd5a and pthlha.
If the mechanical aspects were improved it would greatly strengthen the conclusions of the paper and expand its relevance.

© 2022. Published by The Company of Biologists under the terms of the Creative Commons Attribution License (https://creativecommons.org/licenses/by/4.0/).

First revision

Author response to reviewers' comments

We thank the reviewers for their thoughtful, constructive criticisms and have attempted to address them all, which has greatly improved the paper.

Reviewer 1 Comments for the Author:

1) a more complete expression analysis of pthlha and its receptors in the developing ch is needed (see notes 2 and 3 under General feedback)

We have performed HCR in situs for *pthlha* (together with *ihha* and *sox9a*) at 48, 54, 72, 96 and 168 hours postfertilization (hpf). These confirm our previous results showing localized *pthlha* expression near the ends of the future ch as the cartilage condensation forms and prior to differentiation, a pattern which then does not change much in later larvae. A new Figure 3 shows examples at 48 and 96 hpf, referred to in Results (p. 9, lines 188-192).
We have also performed HCR in situs for *pth1ra* at 72 hpf and although the signal is weak, expression appears widespread and not regionally localized within ch as mentioned in Results (p.9, lines 190-192). We include images for the reviewers, but have not included them in the manuscript. These results are consistent with our model.

2) tests to determine the extent to which mechanical force and Pthlh signaling function separately or are linked during growth plate patterning should be done (see note 6 under General feedback).

We have performed HCR in situs for *pthlha* and *ihha* at 96 hpf in embryos paralyzed by BTX protein injection at 72 hpf. The results show that paralysis disrupts the spatial localization of *pthlha* such that it is more evenly distributed along ch, as well as causing severe reductions in *ihha* expression. These results are shown in a new Figure 8 and referred to in Results (p. 15, lines 428-436).

General feedback:
Introduction:
1. Provide information about entpd5a. What is the function of the gene and why was it looked at? Is it just a marker for pre-hypertrophic chondrocytes or is predicted that its role in phosphate/pyrophosphate metabolism has a functional role in chondrocyte hypertrophy?

It is well known that *entpd5a* expression marks osteoblasts, but we were surprised to find that it marks subsets of developing chondrocytes. Entpd5a functions in skeletal mineralization by regulating phosphate homeostasis and its roles have been studied in the context of osteogenesis in zebrafish, particularly in vertebral formation around the developing notochord. *entpd5a*$^{-/-}$ mutant zebrafish lack bone (Huitema et al., 2012), but potential indirect roles in cartilage hypertrophy have not been addressed. We have added some discussion of this in the Results (p.7, lines 148-150).

Results:
2. In Figure 2, it is very difficult to appreciate that the localization of pthlha expression is restricted to the dorsal and ventral ends of the ch cartilage. Since this is the foundation of the model, consider different images and/or the use quantitative measures. Another option is to use RNAscope to enhance resolution.

Please also see comment 1) above. We have performed HCR in situs for *pthlha* and include new data for 48 and 96 hpf in a new Figure 3.

3. It is proposed that initiation of cartilage hypertrophy is both spatially and temporally regulated by local exposure to pthlh signaling. However, it is unclear what cells the ligand is signaling to. Zebrafish have two receptors: Pth1rA and Pth1rb. In the discussion it is said that pthr1 is expressed ubiquitously throughout embryogenesis (Gray et al ., 2013). This is based on qPCR and in situ experiments between 1-5 dpf that do not distinguish between the two isoforms.

© 2022. Published by The Company of Biologists under the terms of the Creative Commons Attribution License (https://creativecommons.org/licenses/by/4.0/).

The expression pattern of the receptor should be revisited at the stages being studied here using in situ probes that distinguish between the isoforms. This could resolve how pthlh mechanistically acts at a distance in the developing growth plate.

Please also see comment 1) above. We have performed HCR in situs for *pth1ra* and although expression levels are very weak it appears to be expressed throughout the cartilage condensation and surrounding tissue. This is now mentioned in Results (p.9, lines 190-192), and shown in a new Supplementary Figure provided for reviewers that can be combined into the new Figure S1 if necessary.

4. It is said that ihha/b are not expressed, but that Hh signaling is required for expression of the entpda transgene. The authors then say that shh from the endoderm serves as the likely source of Hh. Proximity of shh expression to the cells of interest should be shown to support this idea.

The only published data for *ihha/b* expression are for 120 hpf (Eames et al., 2010). Our HCR in situ data show that both *ihha* and *ihhb* are expressed at 72 and 96 hpf in the pre-HZ, which we include in a new Figure 3 and Figure S1 and describe in Results (p.9, lines 200-201), We detect no expression of either gene earlier at 54 or 60 hpf (data not shown). These results are consistent with our model that localized Pthlh expression precedes Ihh in establishment of the negative feedback loop in the developing growth zone of ch. This is discussed on p.18, lines 486-489.

5. On page 10, it says that CRISPR-induced deletions of pthlha were confirmed in injected embryos by detection of early ossification in entpda+ bone. However, later it is said that BR3, which is an entpda+ bone, was indistinguishable from control. This should be clarified.

Detection of early ossification refers to the bone collar around ch and not BR3. While the osteoblasts that form around ch derive from cartilage, BR3 is a dermal bone that forms independently of cartilage. Since it ossifies at this stage, we used its presence and size to control for developmental delay. We have clarified in text (p.10, lines 273-274; p.12, lines 315-318).

6. Following paralysis, entpda+ pre-hypertrophic chondrocytes in the middle of the ch are lost and, later, proliferating chondrocyte at the ventral region of the ch are significantly reduced. How does this connect with pthlh and Hh signaling and, more specifically, with expression of pthlha, its receptors, and shh in the endoderm? Is the loss of entpda+ cells and decreased cell proliferation secondary to loss of force-regulated pthlh or shh expression? Expression analysis following paralysis would help connect mechanical force and cell signaling in this study.

See also comment 2) above. We have performed HCR in situs for *pthlha* and *ihha* in paralyzed embryos at 96 hpf and show that paralysis disrupts the spatial localization of *pthlha*; It is more evenly distributed along ch. There are also severe reductions in *ihha* expression. These new data suggest that Ihha, as well as possibly Shh, contributes as a source of Hh signals in ch and that changes in numbers of entpd5a+ and proliferating cells are secondary to these changes in gene expression. This is now shown in the new Figure 8 and described in Results (pp. 15-16, lines 428-436).

7. The proliferative zones at the dorsal and ventral tips of the ch seem to form at the interface between pthlh+ cells and pre-hypertrophic/hypertrophic cells. Is this the case? If so, is it then expected that pthlh misexpression would lead to aberrant localization of the proliferative zones after 120 hpf? This would further support the idea that pthlh signaling regulates patterning of the growth plate zones.

We expect changes in Pthlh signal localization to alter the localization of proliferating zones and HZs at later stages. However, we have been unable to test this idea in our experimental system because zebrafish with mosaic misexpression of *pthlha* typically die before two weeks of age, likely because they cannot feed properly. Once they get older there is also insufficient working distance on the confocal microscope to image the ch in living larvae or juveniles.

© 2022. Published by The Company of Biologists under the terms of the Creative Commons Attribution License (https://creativecommons.org/licenses/by/4.0/).

Discussion:
8. In Figure 1 I-L, it is shown that not all entpda+ cells are col10+ in the ch cartilage at 144 hpf. This should be further discussed on page 18, second paragraph. Is it thought that there are there several populations of hypertrophic chondrocytes (entpda+ , col10+, entpda+/col10+)? Or is it believed that entpda+ cells become entpda-/col10+? Are entpda+ hypertrophic chondrocytes thought to be a subpopulation that undergoes trans-differentiation into osteoblasts since entpd5a is traditionally a marker for osteoblasts and involved in mineralization?

This is a really interesting idea. Unfortunately, we have not tested it here as we have not performed long-term lineage tracing. We have added some discussion of this both in the Introduction (p. 3, lines 66-67) and in Discussion (p. 20, lines 539-541).

Specific feedback:
1. Introduction, page 3, second paragraph: It is states that hypertrophic chondrocytes within the growth plate undergo apoptosis. While this has been the dogma for many years, there is now evidence that at least some hypertrophic chondrocytes survive and transdifferentiate into osteoblasts/osteocytes (Yang et al., 2014, PNAS; Yang et al., 2014, Cell Res; Kobayashi et al., 2014, Nature; Jing et al., 2015, JDR; Park et al., 2015, Biology Open)

Suggested changes and selected references have been added.

2. Introduction, second paragraph: The sentence that begins with "This prevents…" should end with a period (delete the comma).

Period added.

3. In Figure 2 legend, please include a statement that entpd5a:killer red+ cells were not found at 48 hpf (similar to what is said in the results section).

Done

4. Supplemental Figure 1 legend: "HZ" is not shown in the figure and should be deleted.

Deleted

5. Figure 3D-E: There are two white arrowheads. While one clearly points to the HZ, it is not clear what the other indicates from the Figure legend.

The second white arrowhead located towards the dorsal side of the ch in panels D and E was meant to indicate expansion of the primary HZ. We have removed it as it seems clear that the primary HZ extends dorsally in the *pthlha* F0 ch depicted in panels D and E and this observation is described in the manuscript text (p. 11, lines 295-297).

6. Figure 3 legend: Remove the second comma between "ch= ceratohyal" and "D =dorsal".

Done

7. Figure 4 legend: "HZ" is not shown in the figure and should be deleted.

Deleted

8. Discussion, page 22: The paragraph that starts with "Chondrocytes may sense force…" is repetitive with the introduction.

This paragraph, now starting on p. 23, has been revised to emphasize the parallel pathways influencing cartilage growth zones in response to force.

9. Discussion, page 23: To what extent does the embryonic origins of endochondral bone (neural crest versus mesoderm) come into play with respect to distinct regulatory mechanisms?

© 2022. Published by The Company of Biologists under the terms of the Creative Commons Attribution License (https://creativecommons.org/licenses/by/4.0/).

We thank the reviewer for this suggestion and have added a sentence discussing this on p. 24 (lines 640-644).

Reviewer 2 Comments for the Author:

The paper is well written, and conclusions are supported by the presented data.

1. *Essential addition*

While the authors do show the expected phenotypic change after CRISPR-Cas9 injection, no successful targeting of the pthlha locus is shown. Since the guides have not been used before, a T7 assay or similar control should be added to show guide efficiency and presence of CRISPR induced cuts.

We now include examples of Heteroduplex Mobility shift Assay (HMA) results for embryos injected with multiplexed *pthlha* gRNAs in new Supplemental Figure 3, panel B. The results show that CRISPR-induced cutting is detected at at least one of the four target sites in every injected embryo. This is described in Results (p. 10, lines 262-263) and in Methods (p.29-30, lines 752-760).

2. *Potential experimental additions*

The analysis of the expression of the b orthologues of ihh and pthlh would complete the analysis of the role of ihh and pthlh in early cartilage patterning. While the authors do describe ihh as a potentially important gene in the establishment of the expression of entpd5 in the pre-HZ, only the expression of ihha is analyzed. Similarly, the expression of pthlhb in early ceratohyal developmental stages has not been analyzed before.

Please see also Comment 1) above. We have performed HCR in situs for both *pthlhb* and *ihhb* at 96 hpf and find that while we do not detect *pthlhb* expression in ch at this stage, *ihhb* expression resembles that of *ihha* and localizes to the HZ. This is now shown in new Supplemental Figure 1 and mentioned in Results on p.9 (lines 192-194 and lines 200-201).

Minor non-experimental changes and additions:

- *Please add which HCR label was used*

We have expanded the methods section to include more details on the amplifiers used in the HCR experiment as well as information on the new HCR probes used to generate new data in the revised manuscript. See Materials and Methods (p. 31, lines 787-791).

- *The addition of a schematic indicating the guide target in the pthlha gene locus would help to clarify the design of the experiment.*

We have added a diagram of the *pthlha* gene indicating the gRNA target sites in new Supplemental Fig 3, panel A.

- *A closeup of the ch in figure 2C-E would make it easier to see the pthlha expression in the ch*

We have added 3 new panels to Figure 2 (F-H) which are magnified views of the ch cartilage outlined in Figure 2 C-E.

Reviewer 3 Comments for the Author:

The paper is well written and easy to follow. The imaging is high quality and the figures are well presented.

In general I have no suggestions for the early sections of the paper which are logical and well done. That said, I think for completeness it would have been good to see what happens in the mosaic expressing zebrafish later in development, i.e. does early misimpression of pthlha lead

© 2022. Published by The Company of Biologists under the terms of the Creative Commons Attribution License (https://creativecommons.org/licenses/by/4.0/).

to continued local disruption to the HZ/ossification with consequences for the patterning of the element in late larval/juvenile fish or is this effect transient?

We agree this would be interesting and we attempted to examine mosaics misexpressing Pthlha at later stages. However, this proved technically difficult since these animals do not survive past two weeks (likely due to an inability to feed). There is also insufficient working distance to visualize transgenic cells in older juvenile live fish. We need more targeted approaches.

The paralysis section feels the most preliminary. As the relationship to mechanical force is a selling point for the paper, and indeed features in the title it feels as though this section would be improved by inclusion of more data for at the very least comparison to other published studies.

We now include more data examining gene expression in response to paralysis. Please see comment 6 in response to Reviewer 1.

While the authors state 'previous studies have shown mechanical forces influence GP dynamics but effects largely interpreted as indirect due to changes in proliferation' I'm not sure this is the case. Work in chick and mouse such as from Paula Murphy and Niamh Nowlan's group have looked at hypertrophy and ossification of chick long bones in relation to paralysis using FE and also in situs for ColX etc (e.g. Nowlan, Murphy, Prendergast J Biomech 2008, Nowlan, Prendergast, Murphy Plos Comp Biol 2008), and other groups too.

It is true that the effects of force in the mentioned studies were not simply interpreted as caused by changes in proliferation, and we have made the relevant changes in the text and added references to reflect this. However, those studies were made in an animal model where proliferation occurs in chondrocytes at the relevant stages. Force has been shown to promote proliferation, and this in turn may lead to an increase in the number of chondrocytes transitioning out of the range of Pthlh and into the HZ. For this reason, an increase in ColX as a result of force may also be, at least in part, due to an increase in the number of hypertrophic chondrocytes leading to a local increase in Ihh levels (it promotes its own expression in the HZ, Chung et al, 1998) and ColX. The lack of chondrocyte proliferation during the relevant stages in zebrafish helps exclude this possibility in order to focus on the effects of force on the Ihh/Pthlh feedback loop.

The paralysis section I think would benefit from comparison with and referencing of other work in which effects of paralysis on cartilage have been studied in fish, including Schwarz and Zelzer's Dev Biol 2012 paper where they showed defects in intercalation and hypertrophy (though they didn't use word hypertrophy - calling it 'stacking' the outlines in their figures show same effects clearly) following MS22 treatment and use of the Nic mutant. There are multiple options the authors could use to follow up and strengthen the mechanical forces section. They could use other drugs (to test whether flaccid vs rigid paralysis have different effects - e..g ones used previously in zebrafish Brunt et al 2016 Osteoathrtis cartilage), could consider FE modeling (e..g Brunt et al J Biomech 2015) or following up with antibodies or in situs for Piezo1/2 Trpv4 (which they themselves mention in the discussion) or looking into Yap/Taz signaling. Ideally one would show the relationship of mechanosensitivity to entpd5a and pthlha. If the mechanical aspects were improved it would greatly strengthen the conclusions of the paper and expand its relevance.

We agree and we are very interested in the genetic aspects of mechanotransduction. We obtained a Trpv4 mutant from another lab and also made one ourselves, but failed to detect any cartilage or growth zone phenotypes. We also used multiplex CRISPR to mutate Piezo1 and Piezo2 individually in entpd5a:kaede transgenic embryos, but these were indistinguishable from controls. Thus, these mechanosensory genes may be partially redundant and we would need to make double mutants. Regarding Yap/Taz, we currently lack tools to manipulate that pathway without causing other non-specific effects, and we do not have a reliable antibody or transgenic animal in our lab to detect changes in the pathway in specific cells. Regarding FE modeling, we would like to use it to address force dynamics in the ch cartilage and how these correlate with the patterning we observe, However, these analyses would take considerable time, which we feel is beyond the scope of the current manuscript.

© 2022. Published by The Company of Biologists under the terms of the Creative Commons Attribution License (https://creativecommons.org/licenses/by/4.0/).

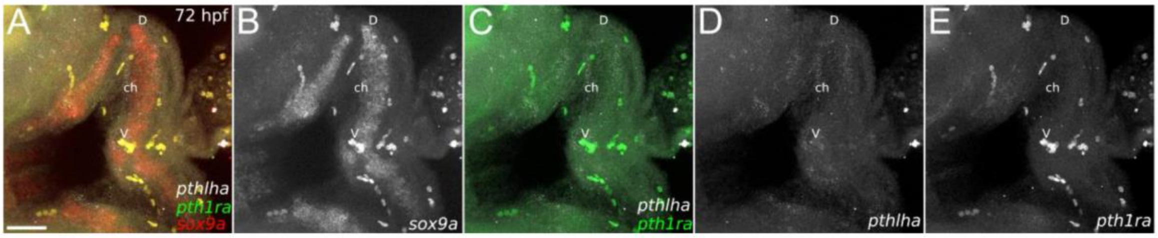

Supplementary Figure X. pth1ra expression at 72 hpf
HCR in situ hybridization for pth1ra, pthlha, and sox9a in wild-type embryos at 72 hpf (A-E). All micrographs are ventral view Z-projections with anterior to the left. ch=ceratohyal, D=dorsal, V=ventral. Scale bar: 50 μm (A-E).

Second decision letter

MS ID#: DEVELOP/2021/199826

MS TITLE: Pthlha and mechanical force control early patterning of growth zones in the zebrafish craniofacial skeleton

AUTHORS: Diego J Hoyle, Daniel Dranow, and Thomas F Schilling

You will be pleased to hear that the referees are happy with your revisions and there are just a few minor issues/typos to consider beforepublication. The referees' comments are appended below, or you can access them online: please go to BenchPress and click on the 'Manuscripts with Decisions' queue in the Author Area.

Reviewer 1

Advance summary and potential significance to field

This exciting study by Hoyle et al. reveals that pthlha signaling and muscle force initiate zonal patterning of the growth plate by specifying pre-hypertrophic chondrocytes within a delimited domain in the cartilage anlage of the ceratohyal (ch) bone in the zebrafish jaw. Novel aspects of this study include the 1) use of entpd5a expression as a novel marker for early pre-hypertrophic chondrocytes and 2) discovery of factors that establish the pthlh-ihh negative feedback loop, which is a key regulator of bone length in development and disease. The authors have carefully addressed all feedback and concerns in this revision.

Comments for the author

Two small errors were found:
1. Page 10, line 215. Replace comma with a period following "ch".
2. Page 13, line 267. "Fig. 4C-F" should be "Fig. 5C-F".

Reviewer 2

Advance summary and potential significance to field

The paper presents a detailed analysis of the role of Pthlha in early patterning of developing cartilages in the zebrafish ceratohyal. The authors identify entpd5a as a marker for the pre-hypertrophic zone (pre-HZ) and show that expression in cartilage cells is depended on pthlha and hedgehog signaling. In addition, they show that the formation of the pre-HZ is dependent on

© 2022. Published by The Company of Biologists under the terms of the Creative Commons Attribution License (https://creativecommons.org/licenses/by/4.0/).

mechanical forces. While a role for Pthlh and mechanical forces in cartilage development has been shown before, the present study for the first time shows the role during the early stages of cartilage patterning.

Comments for the author

The presented additions and revisions to the initial submission have addressed all concerns the reviewers raised and have significantly improved the paper.
There are two minor points the authors might want to address:
1. Could you comment on the large difference of entpd5a positive cells between experiments? At 120 hpf the CyA treatment experiments show 9.9 positive cells in mock treated (page 9) and the numbers observed in the Pthlha knock-out experiment at 120 hpf show 15.6 positive cells in wt controls (page 10). In the BTX-injection experiment, 12.7 positive cells were detected at 96hpf in the control population.
2. On page 7 line 148, I believe this should read ‘correlated with that of col10a1’ instead of col1a1

<u>Reviewer 3</u>

Advance summary and potential significance to field

I am satisfied with the changes made by the authors and am happy to recommend publication.

Comments for the author

I am satisfied with the changes made by the authors and am happy to recommend publication.

Second revision

<u>Author response to reviewers' comments</u>

Point-by-Point Responses to Reviewers

Reviewer 1 Comments for the Author:

Two small errors were found:

1. Page 10, line 215. Replace comma with a period following "ch".

Done

2. Page 13, line 267. "Fig. 4C-F" should be "Fig. 5C-F".

Done

Reviewer 2 Comments for the Author:

There are two minor points the authors might want to address:

1. Could you comment on the large difference of entpd5a positive cells between experiments? At 120 hpf the CyA treatment experiments show 9.9 positive cells in mock treated (page 9) and the numbers observed in the Pthlha knock-out experiment at 120 hpf show 15.6 positive cells in wt controls (page 10). In the BTX-injection experiment, 12.7 positive cells were detected at 96hpf in the control population.

The number of entpd5a-positive chondrocytes can vary not only between experiments but within one clutch of embryos. We are uncertain if this reflects genetic variation or small differences in

© 2022. Published by The Company of Biologists under the terms of the Creative Commons Attribution License (https://creativecommons.org/licenses/by/4.0/).

age. We attempted to use independent staging criteria wherever possible to help control for the latter possibility. Differences between mock-treated and untreated controls may also reflect effects of low levels of EtOH in the mock-treated medium.
Since we do not know the exact cause we have chosen not to modify the text to discuss this variation.

2. On page 7 line 148, I believe this should read 'correlated with that of col10a1' instead of col1a1

That is correct and the text has been modified.

Reviewer 3 Comments for the Author:

I am satisfied with the changes made by the authors and am happy to recommend publication.

Third decision letter

MS ID#: DEVELOP/2021/199826

MS TITLE: Pthlha and mechanical force control early patterning of growth zones in the zebrafish craniofacial skeleton

AUTHORS: Diego J Hoyle, Daniel Dranow, and Thomas F Schilling
ARTICLE TYPE: Research Article

I am happy to tell you that your manuscript has been accepted for publication in Development, pending our standard ethics checks.

© 2022. Published by The Company of Biologists under the terms of the Creative Commons Attribution License (https://creativecommons.org/licenses/by/4.0/).
